# Adipocyte‐specific FAK deletion promotes pancreatic β‐cell apoptosis via adipose inflammatory response to exacerbate diabetes mellitus

**DOI:** 10.1002/ctm2.1742

**Published:** 2024-06-26

**Authors:** Fei Ding, Peng Zheng, Hong‐Ting Fang, Yuan‐Yuan Luo, Xi‐Yue Yan, Hui‐Jian Chen, You‐E Yan

**Affiliations:** ^1^ Department of Pharmacology Wuhan University School of Basic Medical Sciences Wuhan China

**Keywords:** adipose tissue, apoptosis, focal adhesion kinase, inflammatory response, type 1 diabetes, β‐cells

## Abstract

**Background:**

White adipose tissue (WAT) has a key role in maintaining energy balance throughout the body, and their dysfunction take part in the regulation of diabetes mellitus. However, the internal regulatory mechanisms underlying are still unknown.

**Methods and results:**

We generated adipocyte‐specific FAK KO (FAK‐AKO) mice and investigated their phenotype. The cascade of adipocyte, macrophage in adipocyte tissues, and pancreatic β‐cells were proposed in FAK‐AKO mice and validated by cell line studies using 3T3‐L1, Raw264.7 and Min6. The FAK‐AKO mice exhibited glucose intolerance, reduced adipose tissue mass and increased apoptosis, lipolysis and inflammatory response in adipose tissue. We further demonstrate that adipocyte FAK deletion increases β cell apoptosis and inflammatory infiltrates into islets, which is potentiated if mice were treated with STZ. In the STZ‐induced diabetes model, FAK AKO mice exhibit less serum insulin content and pancreatic β cell area. Moreover, serum pro‐inflammatory factors increased and insulin levels decreased after glucose stimulation in FAK AKO mice. In a parallel vitro experiment, knockdown or inhibition of FAK during differentiation also increased apoptosis, lipolysis and inflammatory in 3T3‐L1 adipocytes, whereas the opposite was observed upon overexpression of FAK. Moreover, coculturing LPS‐treated RAW264.7 macrophages with knockdown FAK of 3T3‐L1 adipocytes increased macrophage pro‐inflammatory response. Furthermore, conditioned medium from above stimulated Min6 cells apoptosis (with or without STZ), whereas the opposite was observed upon overexpression of FAK. Mechanistically, FAK protein interact with TRAF6 in adipocytes and knockdown or inhibition of FAK activated TRAF6/TAK1/NF‐κB signaling, which exacerbates inflammation of adipocytes themselves.

**Conclusion:**

Adipocyte FAK deletion promotes both adipocyte apoptosis and adipose tissue inflammation. Pro‐inflammatory factors released by the FAK‐null adipose tissue further trigger apoptosis in pancreatic islets induced by the administration of STZ, thereby exacerbating the diabetes mellitus. This study reveals a link between FAK‐mediated adipose inflammation and diabetes mellitus, a mechanism that has not been previously recognized.

## Graphical abstract

Graphical Headlights

• The FAK suppression of adipocytes causes adipocyte apoptosis and pro‐inflammatory responses to promote M1 macrophage polarisation.

• The FAK suppression of adipocytes activates TRAF6/TAK1/NF‐κB signalling pathway to active inflammatory response.

• FAK mediates crosstalk between adipose and pancreatic tissues, and the FAK suppression of adipocytes promotes pancreatic β‐cell apoptosis.

• The FAK suppression of adipocytes accelerates the pathogenesis of T1D.

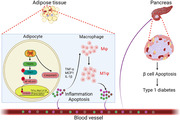

## 1 INTRODUCTION

Diabetes is widespread endocrine system disorder that significantly affects human health. A recent epidemiological survey conducted in 2021 has confirmed an increasing trend in type 1 diabetes (T1D) incidence among individuals aged 0−6 years in Taiwan.^1^ T1D is an autoimmune disease that manifests itself primarily through the damage of pancreatic β‐cells, resulting in reduced insulin secretion.^2^ Diabetes has a complex aetiology, and recent research indicates that pro‐inflammatory cytokines promote pancreatic β‐cell apoptosis.^3^ The study noted the administration of TNF‐α blocker golimumab has significantly enhanced the therapeutic efficacy for diabetic patients.^4^ Hence, investigating the pathways and mechanisms responsible for sources of inflammatory cytokines might be a promising curative strategy for curing diabetes.

Adipose tissue, which comprises many kinds of cells likely adipocytes, endothelial cells and immune cells, is main birthplace of pro‐inflammatory cytokines in bloodstream.^5^, ^6^ Inflammation is closely associated with adipocyte apoptosis, whereby inflammatory cytokines produced by adipocytes promote their own death.^7^ The most numerous immune cells are macrophages in adipose tissue. Upon activation by stimuli such as TNF‐α, macrophages will produce inflammatory cytokines that exacerbate inflammation.^8^ There are complicated crosstalk between adipose tissue and pancreas, and inflammatory factors produced by adipose tissue are the main mediators. Gesmundo et al. reported inflammatory cytokines derived from adipose tissue, as well as extracellular vesicles, contribute to islet β‐cell apoptosis and contributing to the onset of type 2 diabetes (T2D).^9^ However, there have been only a few studies on the effect of adipose tissue on the pathogenesis of T1D.

Focal adhesion kinase (FAK) is a non‐receptor tyrosine kinase and takes part in regulating a variety of biological cellular functions in multiple cancer cells, such as cell proliferation and apoptosis, cell migration and invasion.^10^ The extracellular matrix (ECM) deprivation system showed apoptosis in renal carcinoma cell by repressing FAK expression.^11^ The inhibition of FAK activation through TINK knockdown induces apoptosis in Lung Squamous Cell Carcinoma.^12^ Studies have revealed that FAK expression is the key in the regulation of pro‐inflammatory response. Targeting FAK gene expression could inhibit inflammatory response by suppressing NF‐κB activation in liver cells.^13^ However, there is no complete agreement on the findings of FAK regulation of the inflammatory response. The promotion of pro‐inflammatory transcription factor expression through FAK inhibition mediates anti‐tumour response.^14^ All these studies imply that FAK may have a role in regulating inflammatory response. Previous studies in our laboratory found that HFD‐induced ECM deposition activates FAK/ JNK/ERK1/2 pathway, and promotes adipogenesis in white adipose tissue (WAT).^15^ It suggests that FAK also plays an important function in adipocytes.

Danny has also recently identified the effect of AT inflammation inT1D.^16^ We detected a severe inflammatory signals of AT in adipocyte FAK‐deficient mice. In the non‐obese state, adipocyte FAK‐deficient mice had abnormal glucose tolerance. Therefore, we hypothesised that FAK may modulate the adipocyte inflammatory response and enhance the secretion of inflammatory cytokines, thereby exacerbating diabetes mellitus. Our study investigated FAK of adipocytes for regulating inflammation and its impact on diabetes mellitus. The cascade of adipocyte, macrophage in adipocyte tissues and pancreatic β‐cells were proposed in FAK adipocyte knockout (FAK‐AKO) mice and validated by cell line studies using 3T3‐L1, Raw264.7 and Min6 cells. We provides new evidence for the involvement of adipose tissue in regulating diabetes mellitus, while adipocyte FAK expression could be a new target to treat diabetes.

## 2 MATERIALS AND METHODS

### 2.1 Animal experiment

All experimental animals were on C57BL/6J background. Homozygous FAK floxed mice (*FAK^flox/flox^*) were crossed with Adipoq‐Cre mice to generate FAK AKO mice. *FAK^flox/flox^* mice were used as the wild type (WT). WT and FAK AKO male mice from the same litter were selected for the study. *FAK^flox/flox^* and Adipoq‐Cre mice were purchased from Cyagen Biotechnology Co.(Suzhou, China). All animal experiments are conducted at the Animal Experiment Center of Wuhan University, which has been accredited by the International Association for the Evaluation and Accreditation of Laboratory Animal Care (AUP No. 20210529). Our procedures are based on guidance from the Chinese Commission on Animal Welfare and the International Research Animal Care Council.

For the establishment of Streptozotocin (STZ)‐induced diabetic mouse models, male mice at 8 weeks were selected from both WT and AKO strains. After a 12‐h fast, they received intraperitoneal injections *(i.p.)* of STZ (40 mg/kg HARVEYBIO) for five successive days. Monitoring of blood glucose was every 3 days for 4 weeks after the last injection. The criteria for diabetes in these mice were defined as fasting blood glucose levels exceeding 11.1 mM.^17^ Food intake and water consumption were documented over a period of five consecutive days at 15 days post‐STZ injection. In the normal chow experiments, standard laboratory chow was provided to 6‐week‐old AKO and WT mice for 6 weeks, with body weight being recorded weekly.

In glucose tolerance test (GTT), WT and FAK AKO were administered an *i.p*. of D‐glucose (2 g/kg body weight) after fasting 14 h. Glucose values were recorded at 0, 15, 30, 60 and 120 min following glucose *i.p*. from tail veins. Plasma insulin value were recorded at these time points using the Insulin Elisa Kit to assess insulin secretion. In insulin tolerance test (ITT), mice were injected *i.p*. with insulin (0.75 U/kg body weight) after fasting 4 h, and glucose values were recorded at 0, 15, 30, 60 and 120 min following insulin *i.p*. from tail veins.

For the monitoring of food with water intake, 10 mice were taken from each group, and at 10 weeks, their food with water intake were counted daily and the mean values were calculated. Monitoring was done for five consecutive days.

For body size measurement, mice were anesthetised, and positioned so that their bodies were stretched out on a table. The length of the mouse from the tip of the nose to the anus was read from the scale on the tape measure. For the mouse waist circumference (WC) test, the length of a week at the intersection of the vertical line connecting the mouse raphe to the root of the hind limb. For the mouse body mass index (BMI) test, the weight of the mouse was divided by the square of the body length. Final normalised statistical differences were performed.

$$BMI\;=\frac{{\left(Weight\right)}^{2}}{Body\;length}.$$

### 2.2 Microcomputed tomography

Microcomputed tomography (micro‐CT) imaging (Bruker Biospin MRI GmbH, Skyscan1276, Belgium) was utilised to scan the trunk of mice and reconstruct two‐dimensional drawings into three‐dimensional drawings. Subcutaneous AT is represented in green, while visceral AT is depicted in red.

### 2.3 Comprehensive Animal Metabolic Monitoring System

The Comprehensive Animal Metabolic Monitoring System (CLAMS) system (Columbus Instruments, CLAMS, USA) was used to assess physical activity and energy expenditure of mice, while maintaining a controlled temperature of 25°C and providing sufficient food and water. The CLAX software was employed to analyse the volumes of O_2_ consumption (*VO_2_*), CO_2_ production (*VCO_2_*) and respiratory exchange ratio (RER) during both light and dark periods. Mean values for *VO_2_*, *VCO_2_* and RER were calculated for each mouse.

### 2.4 Histology and immunohistochemistry (IHC)

Pancreas and adipose tissue samples were fixed in 4% paraformaldehyde for 48 h and subsequently embedded in paraffin. The resulting paraffin‐embedded were cut into 4 µm slices (interval of the sections sliced), with the largest cross‐sectional area of each tissue being utilised for haematoxylin‐eosin (HE) staining. HE staining was performed on pancreas sections to assess the insulitis score. The insulitis in each section was graded as follows: 0 for no insulitis, 1 for peri‐islet only, 2 for infiltration within islet less than 50% and 3 for infiltration within islet greater than 50%.^18^ Adipose tissue HE staining was utilised to determine adipocyte area and number. Briefly, import the measurement image and draw a line the same length as the ruler. Next, enter the length of the scale in known distance and ‘µm’ in unit of length, use the hand tool to circle the complete cells in the field of view and measure the area. Count the circled cells and calculate the average cell area in the field of view. For the measurement of the number of fat cells, the fat cells were first modelled as spheres and their radius was calculated from the area, and then their volume was calculated from the radius. According to the density and the weight of AT, the volume of AT was calculated. The number of adipocytes was calculated according to the volume of AT by the volume of individual adipocytes.

To evaluate the islet area and mass, pancreas paraffin sections were immunostained with anti‐insulin (ABclonal, A2090, China) antibody. Adipose tissue paraffin sections were subjected to anti‐F4/80 (CST, 70076S, USA) antibody staining for macrophage quantification. 3,3‐N‐Diaminobenzidine Tetrahydrochloride (DAB) solution (Biosharp, BL732A, China) was used for colour development. Haematoxylin was utilised for nuclear counterstaining, and quantification was conducted using ImageJ software. For islet area measurement, a line of the same length as the scale bar was first drawn, and the ‘Free Select’ tool was used to draw the outline of the islet in the imported picture, and then the ‘Measure’ tool was used to measure the area. To count the β‐cell area, images were first converted to RGB images, and then islet contours were drawn before automatic thresholding. Using the ‘Measure’ tool, the ratio of β‐cell area to islet area can be read.^19^

### 2.5 Cell culture, transfection and co‐culture

#### 2.5.1 3T3‐L1 cell culture, differentiation, transfection and treatment

Design manufacture and engineer management (DMEM) supplemented with 10% Fatal bovine serum (FBS), 1% penicillin and streptomycin (complete medium) was used for cultivation of 3T3‐L1 cells under conditions of 37°C and 5% CO_2_. The preadipocytes of 3T3‐L1 were induced and differentiated into adipocytes by maintaining confluent cells in complete medium, along with insulin (10 µg/mL), dexamethasone (1 µM) and IBMX (0.5 mM) for 2 days from days 0 to 2. Subsequently, the cells were cultured in complete medium and 10 µg/mL insulin for 6 days (Day 2‐Day 8), followed by cell harvesting on Day 8. The process of 3T3‐L1 preadipocyte differentiation into mature adipocytes involves two distinct stages. The entire process spans 8 days, with the first half primarily focused on adipocyte differentiation and the second half on adipocyte maturation. On day 4, transfection of FAK siRNA, FAK overexpression(FAK OE) vector, combining FAK siRNA with TRAF6 siRNA, as well as treatment with a 5‐µM FAK inhibitor (MCE, China), was performed on the 3T3‐L1 adipocytes. The transfection assays were performed by Lipofectamine™ 3000 Reagent (Invitrogen, USA). Transfection methods are summarised below. Dilute 7.5 µL Lipo 3000 in 250 µL DMEM medium and mix. Prepare master mix of 5 µg FAK OE vector or 50 µM siRNA in 250 µL DMEM medium, then add 10 µL P3000™ in FAK OE group, but not siRNA group – mix well. Add diluted FAK OE vector or FAK siRNA to tube of Lipo3000 (1:1 ratio), and incubate for 10−15 min. Add the above complex to differentiated 3T3‐L1 cells. TRAF6 siRNA sequences: Forward 5′‐GCUUGAUGGCAUUACGAGATT‐3′, Reverse 5′‐UCUCGUAAUGCCAUCAAGCTT‐3′. FAK siRNA sequences: Forward 5′‐UGGAUUUGUACCAUUCUUCUG‐3′. Reverse 5′‐GAAGAAUGGUACAAAUCCAAG‐3′.

#### 2.5.2 RAW264.7 cell culture and polarisation

RAW264.7 cells were cultured in complete medium at 37°C and 5% CO_2_. Cells were inoculated into a cell culture chamber (0.4 µm; LABSELECT, China) 1 day prior to stimulation. Afterwards, the cells were stimulated with either 100 ng/mL LPS (Sigma, USA) or 10 ng/mL IL‐4 (SinoBiological, China) for a duration of 48 h.

#### 2.5.3 Co‐culture

Induced differentiation of the aforementioned transfected or treated 3T3‐L1 cells into mature adipocytes (day 8) was conducted in a six‐well plate. RAW264.7 cells were inoculated in a 0.4‐µm cell culture chamber 1 day prior to co‐culture. The two types of cells were co‐cultured and supplemented with either 100 ng/mL LPS or 10 ng/mL IL‐4 for a duration of 48 h. The cell supernatant was collected and centrifuged at 12 000 × g for 10 min to remove cellular fragments.

##### Min6 cells culture and treatment

Min6 cells were cultured in complete medium, 50 µM β‐mercaptoethanol (Gibco, USA) at 37°C and 5% CO_2_. The conditioned medium was prepared by mixing 1 mL of co‐culture cell supernatant with an equal volume of culture medium. Cells were seeded in culture plate (Corning, USA) 1 day prior to treatment and exposed to conditioned medium or treated with 10 mM STZ for 48 h.

### 2.6 Flow cytometry

Single‐cell suspensions were isolated from pancreatic lymph nodes (PLNs) and pancreas obtained from STZ‐induced mice. The cells were stained with the following antibodies: FITC‐CD3 (Elabscience, E‐AB‐F1013C, China), APC‐CD8 (Biolegend, 140419, USA), PE‐CD4 (Elabscience, E‐AB‐F1097D, China), PE‐F4/80 (Elabscience, E‐AB‐F0995G, China), APC‐CD11b (Elabscience, E‐AB‐F1081E, China), FITC‐CD11c (Elabscience, E‐AB‐F0991C, China), APC‐CD206 (Elabscience, E‐AB‐F1135E, China) and APC‐CY7‐B220 (BD, 561037, USA). Single‐cell suspensions of RAW264.7 cells were labelled with PE‐F4/80, APC‐CD11b and FITC‐CD11c antibodies. Single‐cell suspensions of differentiated 3T3‐L1 and Min6 cells were stained using the AnnexinV‐FITC/PI Apoptosis Kit (Elabscience, E‐CK‐A211, China). Visualisation charts were generated on a DxFLEX flow cytometer (Beckman Coulter, cytoflex, Germany) and analysed using FlowJo software.

### 2.7 EdU proliferation assay

To evaluate cellular proliferation, the differentiated 3T3‐L1 adipocytes were stained using the EdU Assay kit (Elabscience, E‐CK‐A376, China). The cell nuclei were counterstained with 4',6‐diamidino‐2‐phenylindole (DAPI). Fluorescence microscopy was employed to assess nucleus‐EDU binding. ImageJ software was utilised to determine the ratio of EDU‐stained nuclei to DAPI‐stained nuclei.

### 2.8 TUNEL apoptosis assay

For the assessment of cell apoptosis, the differentiated 3T3‐L1 adipocytes were stained using the TUNEL Assay kit (Elabscience, E‐CK‐A320, China) to detect DNA fragmentation. To assess apoptosis in pancreas paraffin sections, anti‐insulin antibody and TUNEL reagent were used for staining. We utilised ImageJ software to analyse TUNEL staining exclusively in insulin‐positive cells. For the evaluation of apoptosis in adipose tissue paraffin sections, we employed both anti‐Perilipin 1 antibody (Bioss, bs‐3789R, China) and TUNEL reagent. DAPI was used for nuclear staining. We restricted our analysis of TUNEL staining to only perilipin 1‐positive cells using ImageJ software.

### 2.9 STRING database analysis

Analysis of proteins potentially interacting with FAK was conducted using the STRING database, followed by construction of a protein–protein interaction (PPI) network (https://cn.string‐db.org/). Protein names were selected as search patterns, and ‘Mus musculus’ was set as the organism. By selecting ‘Search’, FAK‐interacting proteins and their scores were obtained; a score greater than 0.9 indicates a strong interaction between two proteins.^20^ Click on the ‘analysis’ option located below the PPI network and proceed to select inflammation‐related pathways with the highest ‘strength’ score for validation in KEGG pathways.

### 2.10 FAK OE vector construction

The 3299 bp FAK was amplified from code DNA extracted from 3T3‐L1 cells as a template for PCR with a pair of gene‐specific primers FAK OE‐F ‘ACCTCTGGATCCACAGAATTCATGGCAGCTGCTTATCTTGACC’ and FAK OE‐R ‘GTCTCCTCTAGATGACTCGAGTCAGTGTGGCCGTGTCTGCCCT’. PCR fragments were amplified and cloned into *EcoR* I and *Xho* I sites of pcDNA3.1 to generate the overexpression vector pcDNA3.1‐FAK by using MultiF Seamless Assembly Mix(Biomarker, RK02003, China).

### 2.11 Real‐time quantitative PCR (qPCR)

Total RNA isolated from cellular or tissue by using TRIzol Reagent (Takara, RNAiso Plus 9108, China) and reverse‐transcribed into cDNA by using PrimeScriptTM RT Reagent Kit (Takara, RR047Q, China). The cDNAs were analysed by qPCR on a CFX96TM Real‐Time PCR Detection System (Bio‐Rad, CA, USA) using SYBR Green qPCR Master Mix (Takara, RR820R, China). Relative gene expression level was determined by the 2^−ΔΔCT^ method and normalised for Rplp0 expression. The primer sequences are listed in Table S1.

### 2.12 Western blot and co‐immunoprecipitation (Co‐IP)

Total protein lysates were extracted from cells or adipose tissue using RIPA lysate (Beyotime, P0013B, China) and phosphatase inhibitors. Immunoblot analysis was performed using a primary anti‐mouse TNF‐α antibody (Beyotime, AF8208, China), anti‐cleaved Caspase 3 antibody (CST, 9664S, USA), anti‐FAK antibody (CST, 3285S, USA), anti‐TRAF2 antibody (ABclonal, A0962, China), anti‐TRAF6 antibody (ABclonal, A0973, China), anti‐phospho‐TAK1 antibody (CST, 9339S, USA), anti‐TAK1 antibody (CST, 5206S, USA), anti‐NF‐κB p65 antibody (CST, 8242S, USA), anti‐phospho‐NF‐κB p65antibody(CST, 3033S, USA) and anti β‐Tubulin antibody (Biopm, PMK181, China). Chemiluminescence signals were detected using ECL solution (HYCEZMBIO, HYC0316, China) and quantified with Image J Software.

For Co‐IP analysis, 3T3‐L1 cells were lysed with RIPA lysate (Beyotime, P0013D, China). A portion of the cell lysate was taken as input while the remaining lysate was incubated with either IgG, TRAF6 or FAK antibodies and then subjected to protein A/G agarose bead precipitation. The resulting protein complexes were collected for subsequent immunoblotting assays.

### 2.13 Enzyme‐linked immunosorbent assay (ELISA)

The concentration of mouse serum insulin was detected using the INS ELISA kit (ELK Biotechnology, ELK1981, China), while the levels of inflammation cytokines in both mouse serum and cell supernatant were determined using TNF‐α ELISA kit (ELK Biotechnology, ELK1387MS, China), IL‐1β ELISA kit (ELK Biotechnology, ELK1271MS, China) and MCP1 ELISA kit (ELK Biotechnology, ELK1271MS, China).

### 2.14 Statistical analysis

The data were presented as mean ± standard error of the mean (SEM). Statistical analyses were performed using GraphPad Prism version 9.4.1, with unpaired t‐test (two‐tailed) and one‐way Analysis of Variance (ANOVA). Statistical significance was considered at *P < 0.05, **P < 0.01, ***P < 0.001.

## 3 RESULT

### 3.1 Adipocyte‐specific FAK knockout (FAK AKO) mice reduced fat mass and impaired glucose tolerance

**FIGURE 1:**
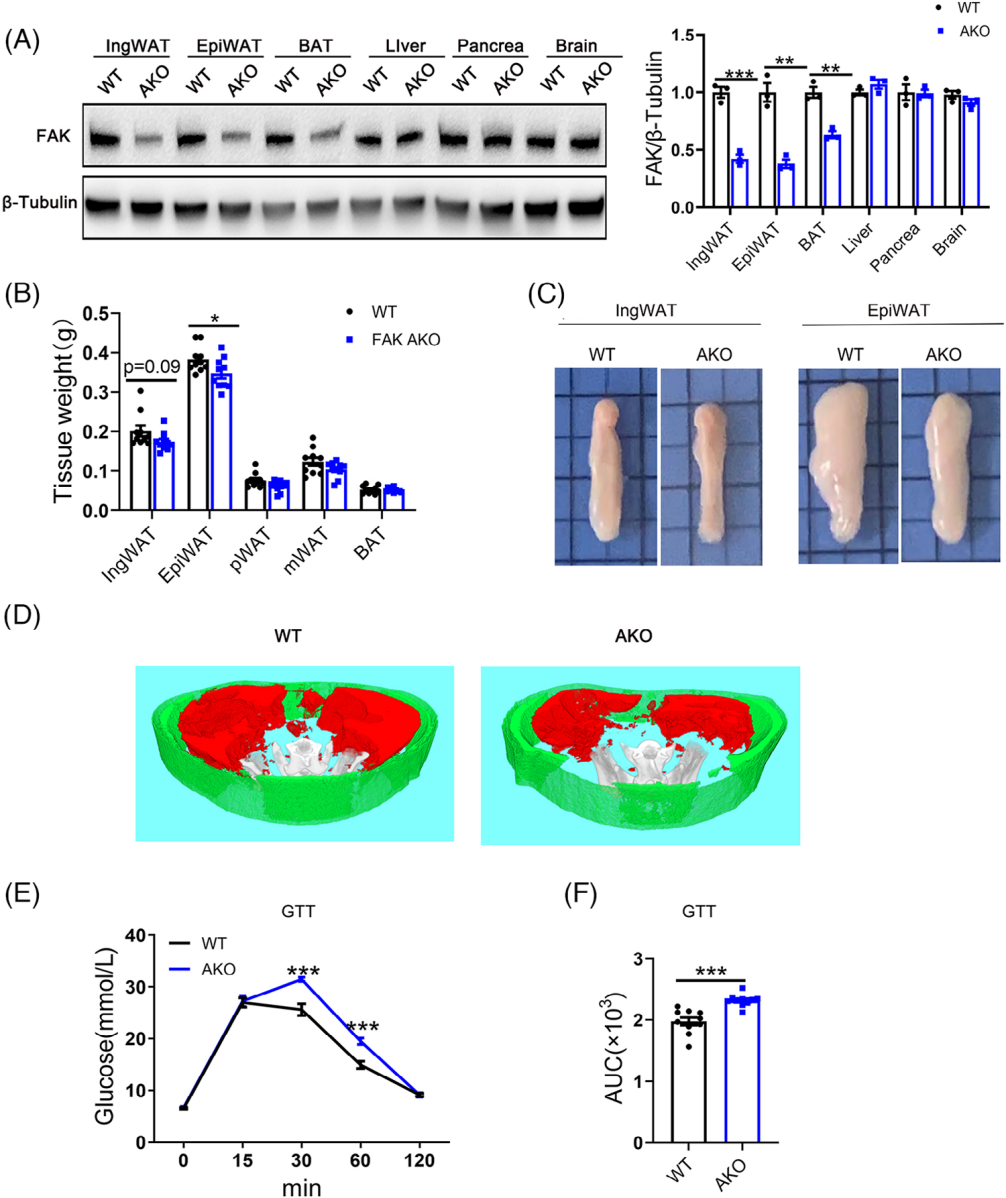
Adipocyte‐specific FAK deletion causes decreased fat mass and abnormal glucose tolerance in mice. (A) Western blot analysis of FAK in the indicated tissues (n  =  5 per group). (B) Weight of adipose tissue at different sites in mice at 12 weeks of age (n  =  10 per group). (C) macroscopic views of IngWAT and EpiWAT. (D) Micro‐CT views of IngWAT and EpiWAT. Subcutaneous fat is represented in green while visceral fat is depicted in red. (E–F) GTT and the AUC of GTT in mice at 10 weeks of age (n  =  10 per group). FAK, focal adhesion kinase; FAK AKO, adipocyte FAK knockout; IngWAT, inguinal white adipose tissue; EpiWAT, epididymal white adipose tissue; pWAT, perirenal adipose tissue; mWAT, mesenteric adipose tissue; BAT, brown adipose tissue; Micro‐CT, microcomputed tomography; AUC, the area under the curve. All values are expressed as MEAN  ±  SEM, ** *P*  <  0.05, *** *P  *<  0.001.

We crossed the Adipoq‐Cre mice with mice carrying a floxed allele of FAK to generate FAK AKO mice. Western blot showed significantly reduction in FAK expression levels in inguinal WAT (IngWAT) and epididymal WAT (EpiWAT) of FAK AKO mice compared with WT, while no discernible changes were observed in other tissues (Figure 1A). Compared to their WT counterparts, FAK AKO mice did not display any differences in terms of body weight, BMI, WC, body size, food and water intake (Figure S1A–E). Compared to WT mice, FAK AKO mice showed a significant reduction in EpiWAT weight and volume, as well as a slight reduction in IngWAT weight and volume (Figure 1B–C). Micro‐CT imaging demonstrated a significant reduction in visceral fat content in FAK AKO mice compared to their WT counterparts (Figure 1D). The GTT results revealed that FAK AKO mice showed significantly higher blood glucose levels than control mice at 30 and 60 min after glucose injection (Figure 1E), with a corresponding increase in the area under the curve (AUC) of GTT observed in FAK AKO compared to WT mice (Figure 1F). No differences were observed in ITT between FAK AKO and control, as well as AUC (Figure S1F–G). The metabolic cage experiments revealed that O_2_ consumption, CO_2_ emission and RER were not different between FAK AKO and control (Figure S2A–C). These findings suggest that FAK AKO mice exhibit weight loss of adipose tissue and impaired glucose tolerance.

### 3.2 Knockout FAK of adipocyte led to an increase adipocyte apoptosis

**FIGURE 2:**
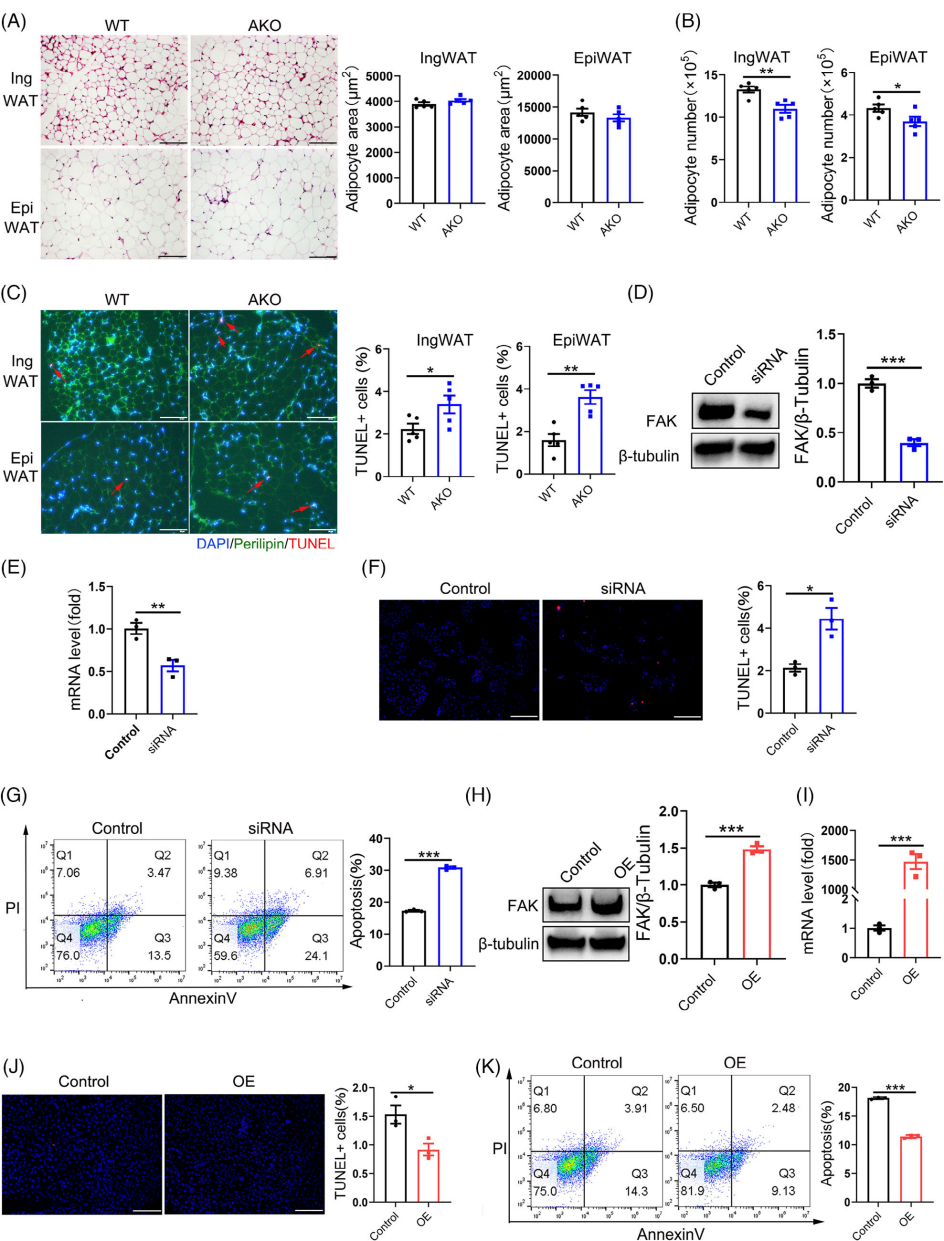
Adipocyte‐specific FAK deletion leads to increased adipocyte apoptosis. (A) HE staining and adipocyte area of IngWAT and EpiWAT (n  =  5 per group). Five different fields of view were counted for each slice and the mean was calculated. Scale bar, 100 µm. (B) The number of adipocyte in IngWAT and EpiWAT (n  =  5 per group). (C) Fluorescence microscope images analysis indicated the TUNEL+ cells(red)in IngWAT and EpiWAT (n  =  5 per group). Five different fields of view were counted for each slice and the mean was calculated. DAPI (blue), perilipin (green) Scale bar, 100 µm. (D–E) qPCR analysis mRNA expression *and* western blot analysis protein expression of *FAK* after transfected FAK siRNA at the day 4 of induced differentiation in 3T3‐L1 cells; samples were collected until day 8 (FAK siRNA group) (n  =  3 per group). (F, J) Fluorescence microscope images analysis indicated the TUNEL+ cells in FAK siRNA or OE group (n  =  3 per group). (G, K) Flow cytometry analysis demonstrated the number of apoptosis cells in FAK siRNA or OE group (n  =  3 per group). (H–I) qPCR analysis mRNA expression *and* western blot analysis protein expression of *FAK* after transfected FAK OE vector at the day 4 of induced differentiation then in 3T3‐L1 cells; samples were collected until day 8 (FAK OE group) (n  =  3 per group). HE staining, haematoxylin‐eosin staining; IngWAT, inguinal white adipose tissue; EpiWAT, epididymal white adipose tissue; OE, overexpression. All values are expressed as MEAN  ±  SEM, *P  <  0.05, **P  <  0.01, ***P  <  0.001.

To investigate the underlying cause of changes in fat mass, we conducted an analysis of adipose tissue morphology in mice. HE staining analysis revealed no discernible differences between FAK AKO and WT mice with respect to adipocyte size from IngWAT and EpiWAT (Figure 2A). However, the number of adipocytes was significantly reduced present in both IngWAT and EpiWAT among FAK AKO mice (Figure 2B). TUNEL staining results revealed that the number of TUNEL‐positive cells was significantly increased in both IngWAT and EpiWAT of FAK AKO mice (Figure 2C). Western blot analysis demonstrated a marked elevation in cleaved caspase3 protein levels within IngWAT and EpiWAT of FAK AKO mice (Figure S3A–B). To further evaluate FAK's role in adipocytes, we transfected FAK siRNA into 3T3‐L1 adipocytes at day 4 of induced differentiation (3T3‐L1 adipocytes). The mRNA and protein levels of FAK exhibited significantly decrease in the FAK siRNA group (Figure 2D–E). TUNEL staining results revealed a significant increase in TUNEL‐positive cells within the FAK siRNA group (Figure 2F). Flow cytometry analysis demonstrated an elevated number of apoptotic adipocytes in the FAK siRNA group (Figure 2G). Western blot indicated a marked increase in cleaved caspase3 protein levels within the FAK siRNA group (Figure S3C). EDU assay findings showed no discernible difference in cell proliferation rates between the FAK siRNA and control groups (Figure S3D). 3T3‐L1 adipocytes were treated with 5 µm FAK inhibitor, yielding results consistent with those observed in the FAK siRNA group (Figure S3E–H). To increase FAK expression levels, overexpression vectors of FAK were transfected into 3T3‐L1 adipocytes. Both mRNA and protein levels of FAK showed significant increases in the FAK OE group (Figure 2H–I). TUNEL staining showed that the number of TUNEL‐positive cells was significantly decreased within the FAK OE group (Figure 2J). Flow cytometry showed a reduction in the number of apoptotic adipocytes in the FAK OE group (Figure 2K). Western blot result showed significantly decrease in cleaved caspase3 protein expression in the FAK OE group (Figure S3I). The EDU assay indicated no difference in cell proliferation ability between FAK OE and control group (Figure S3J).

**FIGURE 3:**
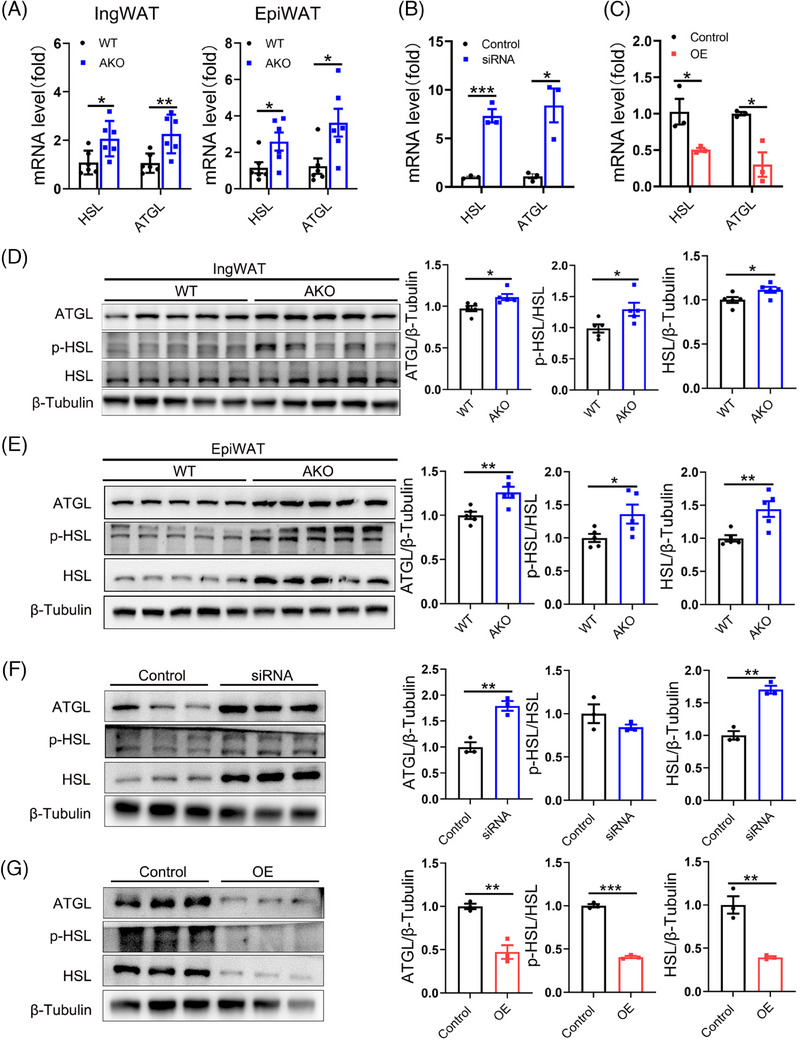
Adipocyte‐specific FAK deletion causes increased lipolysis. (A–C) qPCR analysis mRNA expression of HSL and ATGL in IngWAT and EpiWAT (n  =  6 per group), FAK siRNA group and FAK OE group (n  =  3 per group). (D–G) Western blot analysis indicated protein expression of HSL, p‐HSL and ATGL in IngWAT and EpiWAT (n  =  6 per group), FAK siRNA group and FAK OE group (n  =  3 per group). IngWAT, inguinal white adipose tissue; EpiWAT, epididymal white adipose tissue; All values are expressed as MEAN  ±  SEM, *P  <  0.05, **P  <  0.01, ***P  <  0.001.

qPCR and Western bolt analysis revealed that the levels of HSL, p‐HSL/HSL and ATGL were significantly increased in IngWAT and EpiWAT of FAK AKO mice (Figure 3A, D–E). In vitro experiments further confirmed that knockdown of FAK by siRNA or inhibition by a specific inhibitor led to elevated the levels of HSL, p‐HSL/HSL and ATGL (Figure 3B, F; Figure S3K–L). In contrast, transfection of the FAK OE vector in 3T3‐L1 adipocytes results in a downregulation of lipolytic genes HSL, p‐HSL/HSL and ATGL (Figure 3C,G). These findings collectively suggest that adipocyte‐specific deletion of FAK reduces fat mass by increasing adipocyte apoptosis and promoting lipolysis.

### 3.3 Adipocyte‐specific FAK deletion exacerbated adipose tissue inflammation

**FIGURE 4:**
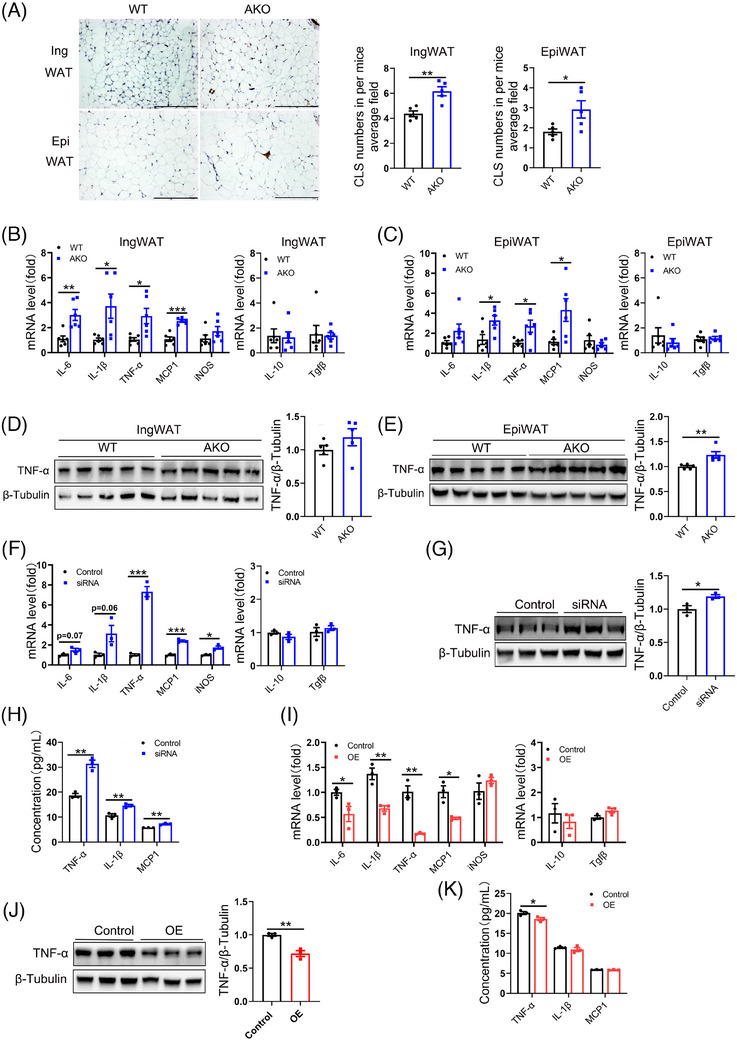
Adipocyte‐specific FAK deletion exacerbates adipose tissue inflammation. (A) IHC staining analysis F4/80 in IngWAT and EpiWAT, as well as quantification of crown‐like structures (CLS) (n  =  5 per group). Five different fields of view were counted for each slice and the mean was calculated. Scale bar, 100 µm. (B–C) qPCR analysis mRNA expression of IL‐6, IL‐1β, TNF‐α, MCP1, iNOS, IL‐10, Tgfβ in IngWAT and EpiWAT (n  =  6 per group). (D–E) Western blot analysis protein expression of TNF‐α in IngWAT and EpiWAT (n  =  5 per group). (F, I) qPCR analysis mRNA expression of IL‐6, IL‐1β, TNF‐α, MCP1, iNOS, IL‐10, Tgfβ in FAK siRNA or OE group (n  = 3 per group). (G, J) Western blot analysis protein expression of TNF‐α in FAK siRNA or OE group (n  = 3 per group). (H, K) ELISA analysis TNF‐α, IL‐1β, MCP1 concentration in the supernatant of FAK siRNA or OE group (n  = 3 per group). IHC, immunohistochemistry; IngWAT, inguinal white adipose tissue; EpiWAT, epididymal white adipose tissue; IL‐6, Interleukin‐6; IL‐1β, interleukin‐1beta; TNF‐α, tumour necrosis factor α; iNOS, inducible nitric oxide synthase; MCP1, monocyte chemoattractant protein 1; IL‐10, Interleukin‐10; Tgfβ, transforming growth factor β; All values are expressed as MEAN  ±  SEM, *P  <  0.05, **P  <  0.01, ***P  <  0.001.

Adipose tissue inflammation is closely associated with adipocyte apoptosis. To investigate the potential involvement of FAK in regulating adipose tissue inflammation, we assessed inflammation‐related markers. Immunohistochemistry results revealed a significant increase in crown‐like structures (CLS) within both IngWAT and EpiWAT of FAK AKO mice (Figure 4A). qPCR result showed significantly increase of inflammatory genes IL‐6, IL‐1β, TNF‐α and MCP1 in IngWAT and EpiWAT of FAK AKO mice, while no difference was detected in the expression of anti‐inflammatory genes (Figure 4B–C). Western blot further confirmed that increase in TNF‐α protein specifically in EpiWAT but not IngWAT of FAK AKO mice (Figure 4D–E). Adipose tissue comprises multiple cell types. Therefore, we utilised adipocyte cell lines to investigate whether FAK regulates the inflammatory response of adipocytes themselves. qPCR analysis revealed that mRNA expression of pro‐inflammatory genes TNF‐α, MCP1 and iNOS was significantly elevated, while IL‐6 and IL‐1β were slightly increased in FAK siRNA group. Conversely, no significant differences were observed in anti‐inflammatory gene levels (IL‐10 and Tgfβ) between FAK siRNA and control group (Figure 4F). Western blot results demonstrated a significant increase in TNF‐α protein expression in the FAK siRNA group (Figure 4G). The Elisa results revealed a significant elevation of TNF‐α, IL‐1β and MCP1 levels in the cell supernatant of the FAK siRNA group (Figure 4H). Consistently, similar observations were made with FAK inhibitor treatment (Figure S4A–C). In contrast, the mRNA level of pro‐inflammatory genes IL‐6, IL‐1β, TNF‐α and MCP1 was a significant reduction in the FAK OE group (Figure 4I), while there was no difference observed in anti‐inflammatory genes. Western blot analysis showed significantly decrease in TNF‐α expression in the FAK OE group (Figure 4J). The Elisa results revealed a significant decrease in TNF‐α levels in the cell supernatant of the FAK OE group (Figure 4K). These findings collectively suggest that FAK AKO exacerbates adipose tissue inflammation, partly due to the inflammatory response produced by adipocytes themselves.

### 3.4 FAK contributed to inflammation by activating TRAF6/TAK1/NF‐κB signalling pathway

**FIGURE 5:**
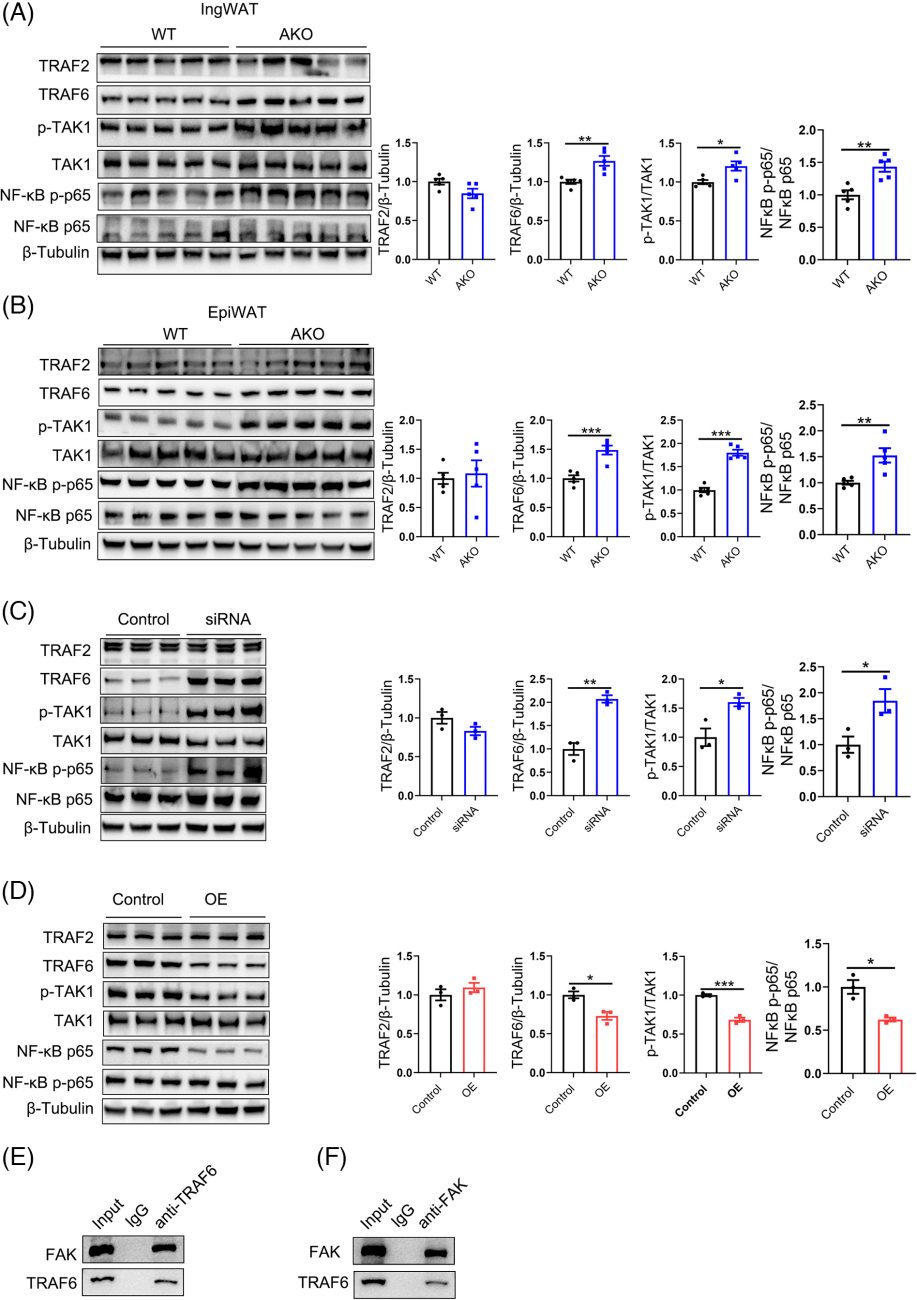
Adipocyte‐specific FAK deletion exacerbates adipocyte inflammatory response through activation of *TRAF6/TAK1/*NF‐κB signalling pathway. (A–B) Western blot analysis protein expression of FAK, TRAF2, TRAF6, TAK1, p‐TAK1, NF‐κB p65, NF‐κB p‐p65 in IngWAT and EpiWAT (n  =  5 per group). (C–D) Western blot analysis protein expression of FAK, TRAF2, TRAF6, TAK1, p‐TAK1, NF‐κB p65, NF‐κB p‐p65 in FAK siRNA or OE group (n  = 3 per group). (E–F) Co‐IP analysis interaction between FAK and TRAF6. IngWAT, inguinal white adipose tissue; EpiWAT, epididymal white adipose tissue; FAK, focal adhesion kinase; TRAF2, tumour necrosis factor receptor‐associated factor 2; TRAF6, tumour necrosis factor receptor‐associated factor 6; TAK1, transforming growth factor‐beta activated kinase 1; NF‐κB p65, nuclear factor kappa‐B p65. All values are expressed as MEAN  ±  SEM, *P  <  0.05, **P  <  0.01, ***P  <  0.001.

To investigate the specific mechanism underlying FAK‐mediated activation of inflammatory response in adipocytes, we performed an analysis of protein that potentially interacts with FAK by using the STRING database (Figure S5A). Subsequently, we performed KEGG signalling pathway enrichment analysis on these interacting proteins and selected the pathway with the highest ‘strength’ score for experimental validation (Figure S5B). We conducted protein assays on the TRAF6/TAK1/NF‐κB signalling pathway. Western blot demonstrated a significant increase in FAK, TRAF6, p‐TAK1/TAK1 and NF‐κB p‐p65/NF‐κB p65 in EpiWAT and IngWAT of FAK AKO mice (Figure 5A–B), while no difference was detected in TRAF2 expression. Similarly, 3T3‐L1 adipocytes results indicated a significant increase in FAK, TRAF6, p‐TAK1/TAK1 and NF‐κB p‐p65/NF‐κB p65 upon FAK siRNA treatment (Figure 5C). FAK inhibitor treatment results were consistent with those observed in the FAK siRNA group, despite no significant difference in NF‐κB p‐p65/NF‐κB p65 levels (Figure S5C). In contrast, Western blot results showed significantly decreased in FAK, TRAF6, p‐TAK1/TAK1 and NF‐κB p‐p65/NF‐κB p65 levels in the FAK OE group (Figure 5D). To evaluate the interaction between FAK and TRAF6, Co‐IP results demonstrated their binding affinity (Figure 5E–F). To further confirm that FAK activates the TRAF6 pathway to regulate inflammatory responses, we constructed FAK and TRAF6 double knockdown 3T3‐L1 adipocytes. Western blot results showed significantly decreased in TRAF6, p‐TAK1/TAK1 and NF‐κB p‐p65/NF‐κB p65 levels in the combining FAK siRNA with TRAF6 siRNA group compared with FAK siRNA group (Figure S5D). These findings suggest that FAK can activate TRAF6/TAK1/NF‐κB signalling pathway to promote inflammatory responses.

### 3.5 Adipocyte‐specific FAK deletion induced polarisation towards M1 macrophages

**FIGURE 6:**
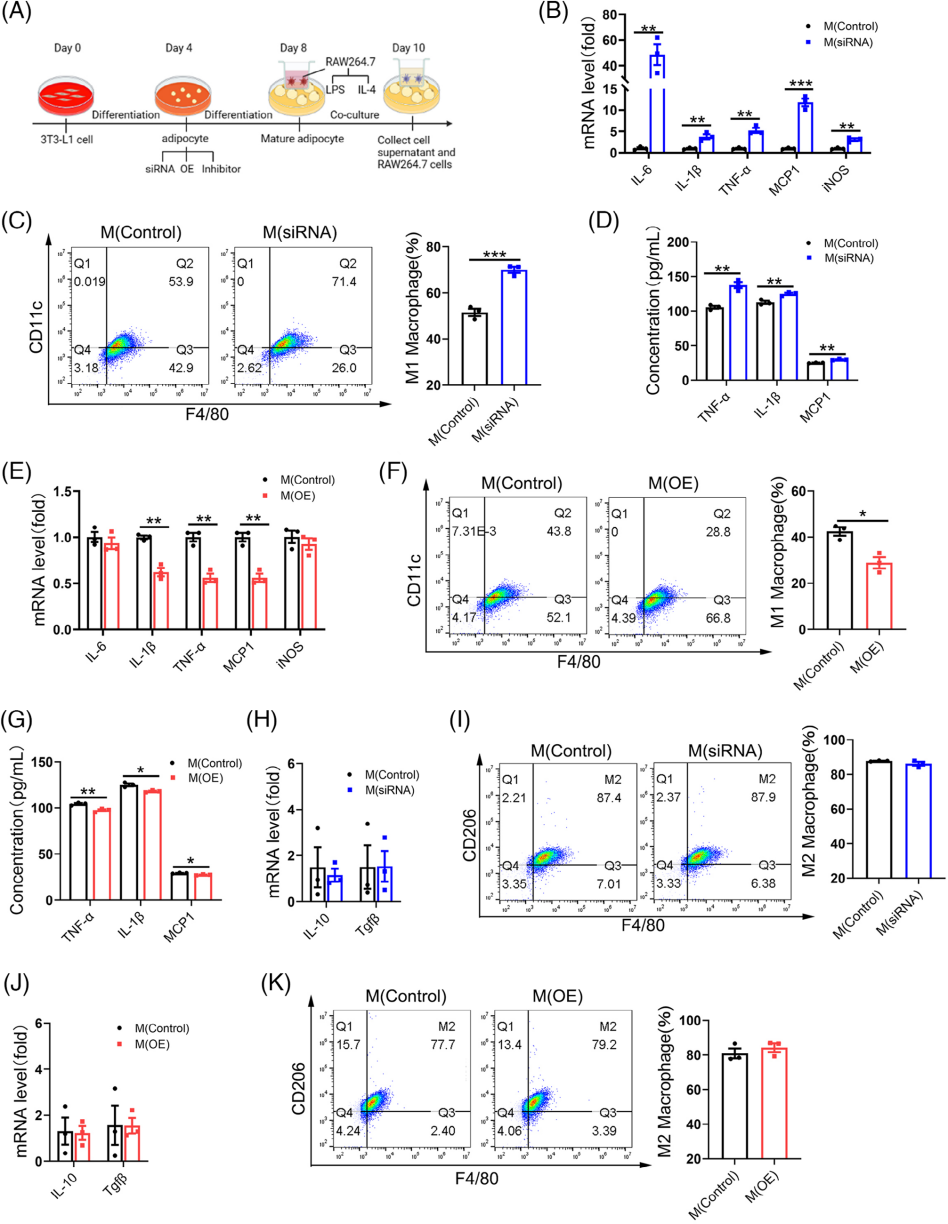
Adipocyte‐specific FAK deletion activate M1 macrophages. (A) Experimental flow chart for co‐culture of macrophages and adipocytes. Macrophages were co‐cultured with adipocytes (transfected FAK siRNA) (B–D) or (transfected FAK OE vector) (E–G) and the addition of LPS stimulation for 48 h. (B, E) qPCR analysis RAW264.7 cells mRNA expression of IL‐6, IL‐1β, TNF‐α, MCP1, iNOS in FAK siRNA or FAK OE group (n  = 3 per group). (C, F) Flow cytometry analysis demonstrated the number of M1 macrophages in FAK siRNA or FAK OE group (n  =  3 per group). (D, G) ELISA analysis TNF‐α, IL‐1β, MCP1 concentration in the supernatant of FAK siRNA or FAK OE group (n  = 3 per group). Macrophages were co‐cultured with adipocytes (transfected FAK siRNA) (H–I) or (transfected FAK OE vector) (J–K) and the addition of IL‐4 stimulation for 48 h (H–I). (H, J) qPCR analysis RAW264.7 cells mRNA expression of IL‐10, Tgfβ in FAK siRNA or FAK OE group (n  = 3 per group). (I, K) Flow cytometry analysis demonstrated the number of M2 macrophages in FAK siRNA or FAK OE group (n  =  3 per group). LPS, lipopolysaccharide; IL‐4, interleukin 4. All values are expressed as MEAN  ±  SEM, *P  <  0.05, **P  <  0.01, ***P  <  0.001.

To investigate the involvement of macrophages in the inflammatory response, co‐culture experiments were conducted by combining adipocytes and macrophages to detect any potential inflammatory phenotypes (Figure 6A), following the methods described previously. Transfected FAK siRNA into 3T3‐L1 adipocytes, which were then co‐cultured with LPS‐induced RAW264.7 macrophages for 48 h, the inflammatory indicators in RAW264.7 macrophages were subsequently detected, revealing significantly increase in mRNA level of pro‐inflammatory genes IL‐6, IL‐1β, TNF‐α, MCP1 and iNOS (Figure 6B). Flow cytometry analysis demonstrated significantly increase in the number of M1 macrophages (F4/80+CD11c+) (Figure 6C). ELISA analysis showed a significant elevation of TNF‐α, IL‐1β and MCP1 concentration in cell supernatant that produced by macrophages combined with adipocytes (Figure 6D). Co‐culture experiments with FAK inhibitor treatment yielded consistent findings as those observed in the FAK siRNA group (Figure S6A–C). In contrast, co‐culturing LPS‐induced RAW264.7 macrophages with 3T3‐L1 adipocytes transfected with FAK OE vector for 48 h caused significantly decrease in mRNA expression of pro‐inflammatory genes IL‐1β, TNF‐α and MCP1 (Figure 6E), as well as a reduction in the number of M1 macrophages (F4/80+CD11c+) according to flow cytometry results (Figure 6F). Additionally, ELISA analysis demonstrated a significant decrease in TNF‐α, IL‐1β and MCP1 levels in cell supernatant (Figure 6G). We subsequently investigated the impact of FAK expression in adipocytes on M2 macrophage polarisation. 3T3‐L1 adipocytes transfected with FAK siRNA were co‐cultured with IL‐4‐induced RAW264.7 macrophages for 48 h, and qPCR analysis revealed no significant differences in mRNA expression of IL‐10 and TGFβ (Figure 6H). Flow cytometry analysis also showed no change in the number of M2 macrophages (F4/80+CD206+) (Figure 6I). The co‐culture results of FAK inhibitor‐treated cells were consistent with those observed in the FAK siRNA group (Figure S6D–E). When 3T3‐L1 adipocytes transfected with FAK OE vector were co‐cultured with IL‐4‐induced RAW264.7 macrophages for 48 h, there was no alteration in the expression of M2 macrophage markers (Figure 6J–K). We further examined the production of inflammatory factors in the combining FAK siRNA with TRAF6 siRNA group. Either adipocyte supernatant or supernatant co‐produced by macrophages and adipocytes, and the concentration of TNF‐α, IL‐1β and MCP1 was significantly decrease in the combining FAK siRNA with TRAF6 siRNA group compared with FAK siRNA group (Figure S6F–G). The aforementioned data suggest that the adipocyte‐specific deletion of FAK not only impacts the adipocyte's self‐inflammatory response but also triggers M1 macrophage activation in adipose tissue.

### 3.6 Adipocyte‐specific FAK deletion induced apoptosis in pancreatic β‐cells

**FIGURE 7:**
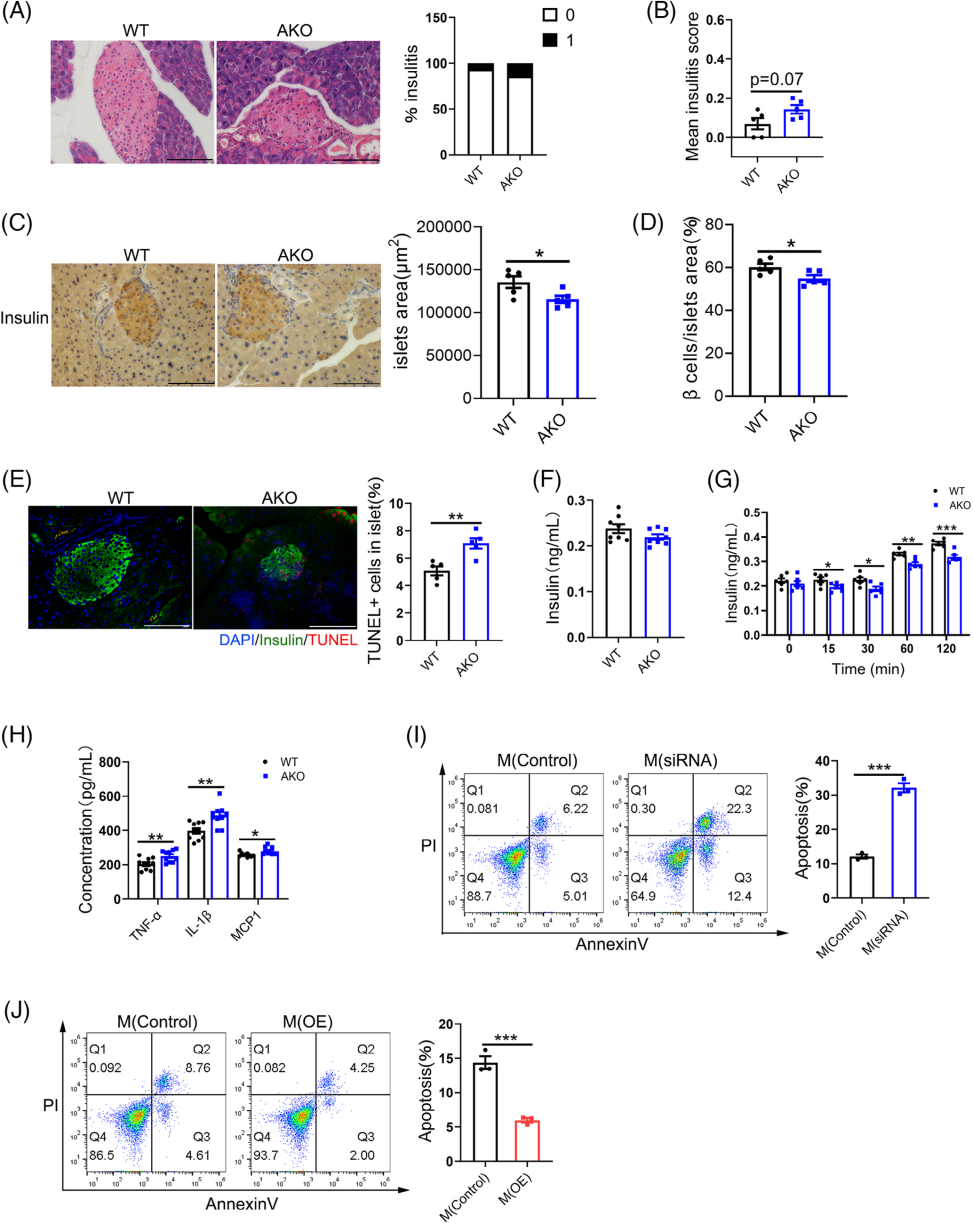
Adipocyte‐specific FAK deletion promote apoptosis of pancreatic β cells. (A) HE staining analysis degree of monocyte infiltration into islets (n  =  5 per group). Scale bar, 100 µm. (B) Calculation of mean insulitis score (n  =  5 per group). (C–D) IHC insulin staining analysis islet area and β cell area over islet area (n  =  5 per group). Scale bar, 100 µm. (E) Fluorescence microscope images analysis indicated the number of TUNEL+ cells in islet (n  =  5 per group). DAPI (blue), insulin (green) Scale bar, 100 µm. (F–G) ELISA analysis serum insulin concentration (n  =  6 or 8 per group). (H) ELISA analysis serum TNF‐α, IL‐1β, MCP1 concentration (n  =  10 per group). (I–J) Supernatant collected from co‐culture of adipocytes (transfected FAK siRNA or FAK OE vector) and macrophages (LPS stimulation) was treated with Min6 cells for 48 h; flow cytometry analysis demonstrated the number of apoptosis cells (n  =  3 per group). All values are expressed as MEAN  ±  SEM, *P  <  0.05, **P  <  0.01, ***P  <  0.001.

Our findings indicate abnormal glucose tolerance in FAK AKO mice, prompting us to investigate the function of their pancreas. HE staining analysis revealed slightly increased in both the number of islets within a score of ‘1’ and mean insulitis score in FAK AKO mice (Figure 7A–B), suggesting potential causes for their impaired glucose tolerance. IHC staining analysis of insulin revealed significantly decrease in the islet area and β‐cell area over islet area in FAK AKO mice (Figure 7C–D). TUNEL and insulin immunofluorescent results demonstrated significantly raise in percentage of TUNEL+β cells over total β cells in FAK AKO mice (Figure 7E). Elisa results revealed that there was no change in serum insulin concentration both FAK AKO and control before addition of glucose (Figure 7F). However, the serum insulin concentration of FAK AKO mice was a significant lower than control after glucose injection (Figure 7G). Elisa analysis showed significantly increase in serum TNF‐α, IL‐1β and MCP1 concentrations in FAK AKO mice (Figure 7H). To further validate these findings in vivo, an In vitro experiment was conducted using cell co‐cultured supernatant from transfected FAK siRNA of 3T3‐L1 adipocytes and LPS‐induced RAW264.7 macrophages to treat the β‐cell line Min6. Compared to control group, the results of flow cytometry indicated a significant increase in apoptosis of Min6 cells (Figure 7I). The co‐culture experiments with FAK inhibitor treatment were consistent with the observations made in the FAK siRNA group (Figure S7A). In contrast, when we treated β‐cell line Min6 with cell co‐cultured supernatant collected from transfected FAK OE vector of 3T3‐L1 adipocytes and LPS‐induced RAW264.7 macrophages, flow cytometry analysis demonstrated significantly decrease in the apoptosis of Min6 cells (Figure 7J). We further examined the apoptosis of 3T3‐L1 adipocytes and Min6 cells in the combining FAK siRNA with TRAF6 siRNA group. Flow cytometry analysis demonstrated an decreased number of apoptotic adipocytes in the combining FAK siRNA with TRAF6 siRNA group compared with FAK siRNA group (Figure S7B). 3T3‐L1 adipocyte supernatants were collected to treat Min6 cells. Flow cytometry analysis demonstrated an decreased number of apoptotic adipocytes in the combining FAK siRNA with TRAF6 siRNA group compared with FAK siRNA group (Figure S7C). 3T3‐L1 adipocytes and macrophage co‐culture supernatants were collected to treat Min6 cells. Flow cytometry analysis demonstrated an decreased number of apoptotic adipocytes in the combining FAK siRNA with TRAF6 siRNA group compared with FAK siRNA group (Figure S7D). The aforementioned data suggested that pro‐inflammatory cytokines generated by adipocyte‐specific FAK deletion promote pancreatic β‐cell apoptosis via the bloodstream.

### 3.7 Adipocyte‐specific FAK deletion exacerbated the incidence of T1D

**FIGURE 8:**
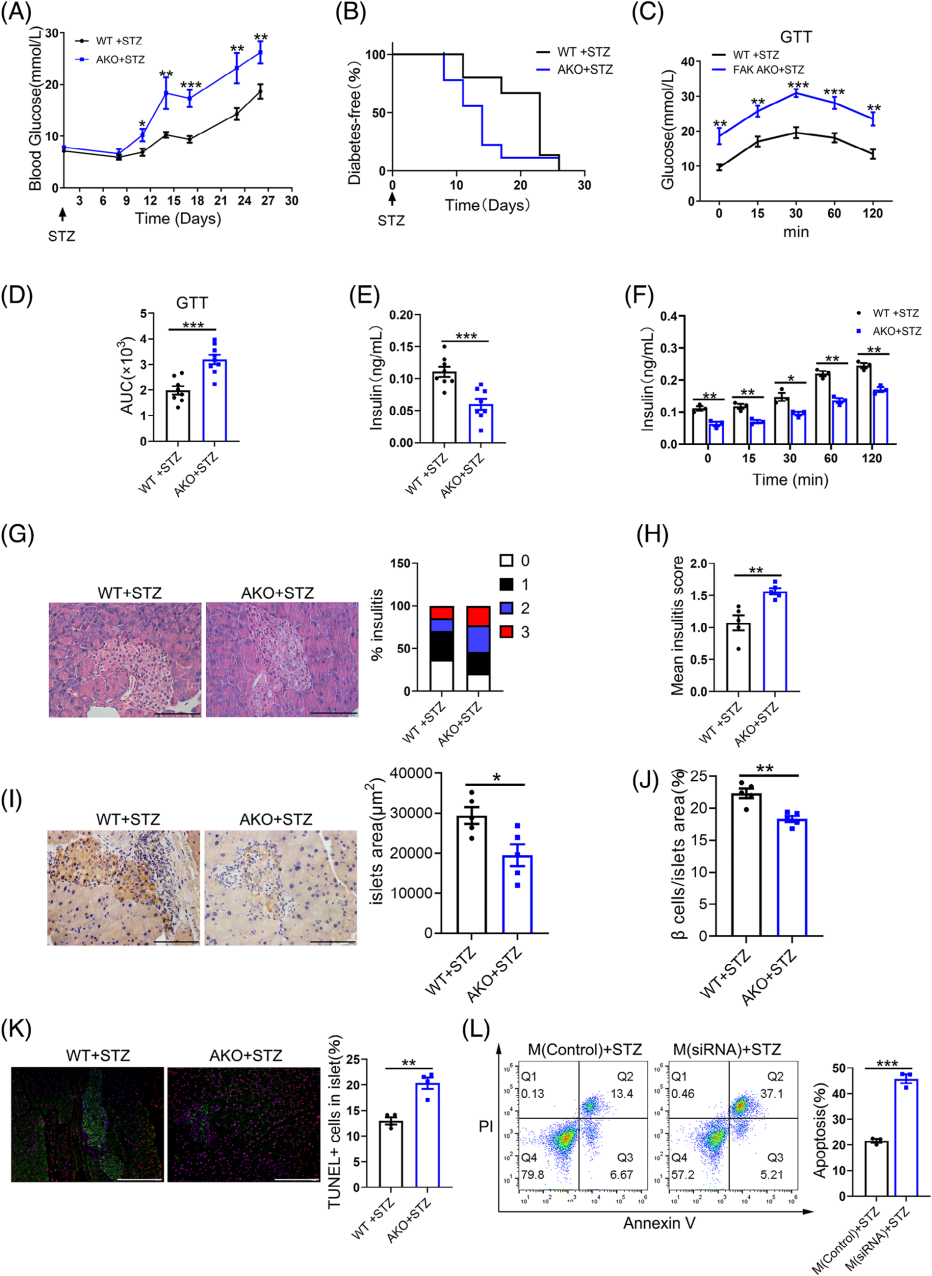
Adipocyte‐specific FAK deletion exacerbates T1D incidence. Mice were injected with 40 mg/kg/day of STZ for five consecutive days. (A) Blood glucose levels at the indicated time points (nWT = 16, nAKO = 9). (B) Incidence of diabetes (nWT = 16, nAKO = 9). (C–D) GTT was performed at day 21 and statistical AUC (nWT = 8, nAKO = 9). (E–F) ELISA analysis serum insulin concentration (n  =  8 per group). (G) HE staining analysis indicated degree of monocyte infiltration into islets (n  =  5 per group). Five different fields of view were counted for each slice and the mean was calculated. Scale bar, 100 µm. (H) Calculation of mean insulitis score (n  =  5 per group). (I–J) IHC insulin staining analysis islet area and β cell area over islet area (n  =  4 per group). Five different fields of view were counted for each slice and the mean was calculated. Scale bar, 100 µm. (D) Calculation of β cell mass from pancreas weight and percentage of β cell area over pancreatic area(n  =  4 per group). (K) Fluorescence microscope images analysis indicated the number of TUNEL+ cells in islet (n  =  4 per group). (L) Supplementation of supernatant collected from co‐culture of adipocytes (transfected FAK siRNA) and macrophages (LPS stimulation) with STZ was treated with Min6 cells for 48 h. Flow cytometry analysis the number of apoptosis cells (n  =  3 per group). STZ, Streptozotocin. All values are expressed as MEAN  ±  SEM, *P  <  0.05, **P  <  0.01, ***P  <  0.001.

To assessment the influence of FAK on adipocyte‐induced pancreatic β‐cell apoptosis and its effect on diabetes mellitus, we prepared an *STZ‐induced* diabetic mouse model. From day 11, fasting blood glucose levels in STZ‐induced FAK AKO was obviously higher than STZ‐induced control (Figure 8A). The morbidity of diabetes was higher in STZ‐induced FAK AKO mice (Figure 8B). Body weight was reduced in diabetes model of FAK AKO mice (Figure S8A). Food and water intake were a significant increase in T1D model of FAK AKO mice (Figure S8B–C). GTT results demonstrated that the blood glucose levels at various time points and AUC of STZ‐induced FAK AKO mice were significant higher than control (Figure 8C–D). Elisa results indicated a significant decrease in serum insulin concentration (Figure 8E), while TNF‐α, IL‐1β and MCP1 were increased in STZ‐induced FAK AKO mice (Figure S8D). The serum insulin concentration of STZ‐induced FAK AKO was lower than that control at various time points after glucose injection (Figure 8F). HE staining analysis revealed slightly increase in the number of islets with a score of ‘2’ or ‘3’ and mean insulitis score in STZ‐induced FAK AKO mice (Figure 8G–H). IHC staining of insulin analysis revealed significantly decrease in the islet area and β‐cell area over islet area in STZ‐induced FAK AKO mice (Figure 8I–J). TUNEL and insulin immunofluorescent results showed a significant increase in the percentage of TUNEL‐positive β‐cells over total β‐cells in STZ‐induced FAK AKO mice (Figure 8K). The degree of immune cell infiltration in the pancreas may reflect the progression of T1D. Flow cytometry results showed significantly increase in the number of CD4+T(CD3+CD4+) and CD8+T(CD3+CD8+) cell, while M1 macrophages (F4/80+CD11b+) remained unchanged in the PLN of STZ‐induced FAK AKO mice (Figure S8E–G). Moreover, there was a marked elevation in the number of CD4+ T cells, B cells(B220+) and M1 macrophages with a slight increase observed for DC cells (CD11b+CD11c+), and no difference was noted for CD8+T cell numbers in the pancreas of STZ‐induced FAK AKO (Figure S8H–L). In vitro, we collected supernatant from co‐cultured cells of transfected FAK siRNA in 3T3‐L1 adipocytes and LPS‐induced RAW264.7 macrophages, supplemented with STZ to treat the β‐cell line Min6. Compared to control group, flow cytometry analysis indicated significantly increase in apoptosis of Min6 cells (Figure 8L). These findings collectively demonstrate that adipocyte‐specific deletion of FAK exacerbates diabetes incidence.

## 4 DISCUSSION

Adipose tissue is crosstalked with several organs in the body, leading to the development of several metabolic diseases, such as diabetes mellitus.^21^ However, the molecular mechanisms by which these interactions occur are still unknown. The currently accepted evidence suggests that adipose tissue‐induced inflammatory responses are one of major contributor to metabolic diseases.^22^ Adipose tissue metabolism disorders have effects on glycolipid metabolism, cytokine and adipokine secretion.^23^ Disorders of glycolipid metabolism and abnormal secretion of lipokines induce systemic inflammation, suggesting that inflammatory cytokines of WAT may be an important regulator for metabolic diseases. It is reported that inflammatory cytokines exacerbate diabetes mellitus by disrupting pancreatic β‐cell survival.^24^ Recent studies have reported that visceral adipose tissue inflammation exacerbates T1D pathogenesis.^16^ In spite of this interest, the molecular mechanisms linking WAT inflammatory response disruption to β‐cell survival are not completely known.

FAK‐associated studies have focused mainly on cancer, and the FAK expression in cancer has been associated with poor outcome.^25^ However, FAK has been less studied in other cell types. Our previous study found that the high expression of FAK occurred in obese humans and mice, and activation FAK/JNK/ERK1/2 signals promoted adipogenesis.^15^ These results suggest that the FAK may be a novel target for disease treatment in adipose tissue. In our research, we further use adipocyte‐specific FAK knockout (FAK‐AKO) that demonstrates relationship between adipose inflammation and the progression of diabetes. We found that loss of adipocyte FAK results in glucose intolerance, reduced adipose tissue mass and increased apoptosis and inflammatory response in adipose tissue. Our study confirmed that adipocyte selective loss of FAK increases beta cell loss and inflammatory cell infiltrates into islets. Importantly, pro‐apoptotic inflammatory factors released by the FAK‐null adipocytes that either trigger apoptosis in islet β‐cells or exacerbate the apoptotic phenotype induced by the administration of STZ. Our result evidence that WAT inflammation and apoptosis linked to beta cell function and the progression of diabetes by adipocyte FAK expression.

Impaired pancreatic islet function is closely related to the development of diabetes.^26^ Liu et al. reported that FAK affects the expression of glucose metabolism‐related genes by activating the PI3K/AKT signalling pathway.^27^ Lu et al. discovered that FAK signalling in adipocytes regulates the development of fatty liver.^28^ These results indicate that FAK plays an important function to regulate metabolic diseases. Being consistent with prior observations, in our study, the FAK AKO mice exhibited abnormal glucose tolerance along with decreased fat. After glucose injection, the serum insulin concentration of FAK AKO was significantly lower than control. This result shows that mice have impaired islet function and decreased insulin production capacity. Luk et al. found that GTT did not change significantly in FAK AKO mice at 24, whereas fasting blood glucose was significantly elevated in FAK AKO mice at younger ages.^29^ Our results were coincided with this observation (younger FAK AKO mice exhibit abnormal glucose metabolism). It is well established that T1D patients are more likely to be occur in the young population.^30^ Combining our findings with those of Luk et al., age differences as a major factor contribute to abnormal glucose metabolism in FAK AKO mice. We further found that 8‐weeks FAK AKO mice had significantly higher blood glucose levels and shorter onset cycles than WT mice in the injected STZ‐induced diabetes model. In addition, FAK AKO mice had deteriorated glucose clearing efficiency and the serum insulin level was significantly reduced after glucose injection in STZ‐induced diabetes model. Thus, it is convinced that FAK AKO mice have increased diabetes susceptibility in young adulthood adipocyte‐specific FAK deletion causes disruption of glucose metabolism and exacerbates the development of diabetes in mice.

Inflammatory response caused by adipose tissue is one of the main factor leading to metabolic diseases.^22^ Jae‐Young Park et al. demonstrated that FAK regulates the inflammatory response of vascular endothelial cells in inflammatory diseases such as slow‐onset atherosclerosis.^31^ Jin et al. found that in diabetic cardiomyopathy, FAK is involved in regulating chronic inflammation in cardiomyocyte cardiomyocytes.^32^ Therefore, FAK is an important regulator of inflammatory responses in a variety of metabolic diseases. Luk et al. reported that the deletion of FAK in adipocytes was able to cause an inflammatory response and adipocyte apoptosis in adipose tissue.^29^ This is consistent with our findings. On the basis of these findings, we further observed CLS and increases of MCP1 and pro‐inflammatory cytokines (TNF‐α and IL‐1β) in WAT of FAK AKO mice. Using 3T3‐L1 cells, we also found that knockdown or inhibition of FAK during differentiation also increased inflammatory gene expression, whereas the opposite is observed upon overexpression of FAK. Additionally, the release of TNF‐α and MCP1 from adipocytes stimulates pro‐inflammatory cytokine secretion by adipose tissue‐resident macrophages (ATMs), including IL‐6, TNF‐α, CCR2 and iNOS.^33^ We also confirmed that TUNEL+ cells and cleaved caspase3 protein were induced in the WAT of FAK AKO mice. Use of various techniques to knock down, inhibitor or overexpress FAK In vitro further corroborated the in vivo findings. Adipocyte apoptosis and inflammation exacerbate M1 macrophage polarisation in adipose tissue.^34^ We further found that co‐culture with adipocytes knocking down or inhibiting FAK expression increased macrophage polarisation to M1 type and increased the pro‐inflammatory gene expression (TNF‐α, IL‐1β, MCP1). In the study of Luk et al., the absence of FAK in adipocytes was able to induce adipocyte apoptosis and lead to adipose tissue inflammation, and on this basis, we further found that FAK deficiency in adipocytes induced both inflammatory response and apoptosis in adipocytes, and that adipocytes activated M1 macrophages in AT through the release of a variety of inflammatory factors to exacerbate the inflammation of AT. Adipocyte apoptosis and inflammatory responses are usually accompanied with increased lipolysis.^35^ Our further research demonstrated that the lipolytic protein expression of HSL and ATGL was increased in WAT of FAK AKO mice. The lipolytic protein level of HSL and ATGL was increased in knockdown or inhibition of FAK expression in adipocytes. However, we detected only a slight decrease in the WAT weight of FAK AKO mice and did not change body weight, which may be due to an increase in compensatory differentiation of adipose stem cells, and this warrants further investigation. Therefore, FAK deficiency in adipocytes both promotes inflammatory responses and apoptosis in adipocytes, and this process activates activation of M1‐type macrophages, exacerbating the inflammatory response in adipose tissue.

Mechanistically, we investigated the aetiology of the inflammatory response. After drug treatment of cervical cancer cells, FAK expression was accompanied by down‐regulation of NF‐κB p65, but the exact regulatory mechanism was not clear.^36^ NF‐κB is widely recognised as a prototypical pro‐inflammatory signalling pathway.^37^ The canonical cascade of the NF‐κB pathway requires activation of TAK1 first,^38^ which has been demonstrated to be associated with adipocyte survival.^39^ A review of the literature showed that TRAF‐interacting proteins associated with the tumour necrosis factor receptor increase NF‐κB expression by activating TAK1.^40^ Interestingly, TRAF6 facilitates the translocation of the NF‐κB dimer from the cytoplasm to the nucleus by promoting TAK1 phosphorylation and hence up‐regulated pro‐inflammatory genes level, likely TNF‐α.^41^ We used the STRING website to find that FAK has direct interaction with TRAF6 protein and activated the NF‐κB inflammatory signalling pathway. Specifically we observed that interaction between FAK and TRAF6 protein. Moreover, the levels of TRAF6, p‐TAK1 and NF‐κB p‐p65 were increased in WAT of FAK AKO mice. In vitro, use of various techniques to knock down FAK further corroborated the in vivo findings. We also used TRAF6 and FAK double knockout adipocytes to confirm that FAK regulates inflammatory responses through TRAF6. Consequently, adipocyte FAK knockdown is able to activate TRAF6/TAK1/NF‐κB signalling, which exacerbates the inflammatory response of adipocytes themselves and secretion of proinflammatory cytokines.

Researches report that crosstalk existed between the pancreas and adipose tissue. Researchers initially believed that the pancreas regulates adipose tissue function in a unidirectional manner and that insulin inhibits lipolysis.^42^ Conversely, inflammatory cytokines released from adipose tissue, such as TNF‐α and IL‐1β, increase islet β‐cell apoptosis. However, the regulatory mechanisms are complex and not fully understood. Our findings indicate that FAK AKO mice exhibit increased serum levels of pro‐inflammatory cytokines (TNF‐α, IL‐1β and MCP1) along with an increase in pancreatic β‐cell apoptosis. Moreover, pancreatic β‐cell area and glucose‐stimulated insulin secretory capacity were decreased, and insulitis scores were slightly increased in FAK AKO mice. Further studies revealed that knockdown or inhibition of FAK expression in adipocytes increased the inflammatory factors concentration (TNF‐α, IL‐1β and MCP1) in cell supernatants. In addition, co‐culturing the above cells with macrophages resulted in similar changes in levels of the inflammatory factors in the cell supernatants. Treatment of pancreatic islet β‐cells with supernatants from knockdown FAK adipocytes co‐cultured with macrophages resulted in increased apoptosis. Blocking the TRAF6 pathway in adipocytes was able to reverse the inflammatory response and apoptosis induced by FAK deficiency. In addition, we also detected increased levels of inflammatory factors in the serum of FAK AKO mice. Denny's research found that the release of inflammatory factors in visceral adipose tissue exacerbated pancreatic beta cell apoptosis and diabetes pathogenesis.^16^ These conclusions were consistent with our findings. Consequently, the pro‐inflammatory factors generated by the adipocytes lacking FAK travel through the blood stream to reach the pancreas (where they induce apoptosis in beta‐cells). It is reported that β‐cell apoptosis is a major cause of abnormal glucose tolerance.^43^ Therefore, we believe that the abnormal glucose tolerance observed in FAK AKO mice is attributed to increased apoptosis of islet β‐cells mediated by inflammatory factors released from adipose tissue. Interestingly, it was no different in serum insulin concentration in the fasting state in FAK AKO and WT mice. However, serum insulin levels were lower in FAK AKO than control after glucose *i.p*. These results indicate a significant reduction in insulin secretion capacity of FAK AKO mice under glucose stimulation, highlighting the importance of FAK as a mediator for crosstalk between adipose and pancreatic tissues.

The association between pancreas and adipose tissue implicates the crosstalk of many cells types and associated secretory phenotype (SASP). In adipose tissue, localised hypoxia‐stimulated mitochondrial ANT2 activates the expansion of a translational factor, hypoxia‐inducible factor 1α (HIF1α), which leads to the secretion of chemokines to entice macrophages. The metabolism of these macrophages shifts from fatty acid oxidation to glycolysis, and their polarisation is shifted to M1 phenotype, which triggers AT remodelling.^44^, ^45^ This leads to increased levels of circulating inflammatory factors. When cytokines like IL‐1 and tumour necrosis factor (TNF) levels are increased, the cytokines bind to cytokine receptors on the surface of β‐cells leading to β‐cell apoptosis.^46^, ^47^ In our study, FAK deletion in adipocytes leads directly to adipocyte apoptosis, and we similarly detected high levels of a combination of pro‐inflammatory factors in mouse serum. This may lead to short‐term pancreatic beta compensatory proliferation, but with such a high concentration of inflammatory factor combinations, this quickly switches to stimulate β‐cell apoptosis. And this is confirmed by recent studies that adipose tissue plays an important influence in the pathogenesis of T1D.^16^ Moreover, in the present study, multiple factors took part in the mechanism of islet β‐cell apoptosis caused by FAK AKO mice; nevertheless, inflammatory factors released by adipose tissue inflammation may be the key factor. In addition to inflammatory factors, free fatty acids (FFAs) are lipotoxic to the pancreas. In the present study, the absence of FAK in adipocytes increased expression of the lipolysis‐related proteins HSL and ATGL, which inevitably produces a large amount of (FFA) reaching the pancreas through the circulation, thus producing pancreatic lipotoxicity and affecting β‐cell apoptosis. Adiponectin and leptin, the classical adipokines produced by adipocytes, also play key roles in the regulation of islet β‐cell function. Some studies have reported that adiponectin was able to stimulate insulin secretion by inactivating insulin and up‐regulating the expression of the insulin gene.^48^ Leptin has been shown to protect beta cells from cytokines‐induced apoptosis.^49^ In the present study, FAK AKO mice had a reduced number of adipocytes. Decreased adipocyte numbers necessarily lead to decreased secretion of lipocalin and leptin, which may further affect β‐cell function. This needs to be further explored. Taken together, adipose‐selective loss of FAK slightly increases β‐cell loss and inflammatory cell infiltrates into islets, which stimulate apoptosis of pancreatic β‐cells, may be contribute to the diabetes mellitus.

Diabetes are considered to be a proinflammatory state.^50^, ^51^ The mechanisms by which inflammatory factors in the circulation regulate βcell apoptosis are complex; it is possible that inflammatory factors directly cause beta cell apoptosis, or that beta cell apoptosis occurs as a result of activation of the innate immune response within the pancreas by inflammatory factors. Pro‐inflammatory cytokine may trigger the enrichment and activation of innate immune cells, leading to β‐cell apoptosis.^52^, ^53^, ^54^ It is now generally accepted that cytotoxic T cells are the main immune cells responsible for the pathogenesis of T1D, and that only effector T cells cause apoptosis of β‐cells by recognising the T‐cell receptor (TCR).^45^ A new perspective has recently suggested that the targeting of macrophages in the pancreas as a therapeutic target for T1D has important clinical implications. This is due to the fact that macrophage clearance from the pancreas significantly reduces T cell entry into the islets and ultimately results in a failure of antigen presentation.^55^ Our further research found an increased number of macrophages, CD4+ T and B cells in the pancreas of FAK AKO mice. In addition, there was an increase in CD4+ T, CD8+ T cells in the PLN. Based on the above results, we believe that the increased incidence of diabetes due to adipocyte FAK deficiency, in addition to the direct stimulation of pancreatic β‐cell apoptosis by inflammatory factors produced by adipose tissue, may also be due to the activation of immune cells such as T cells in the pancreas by inflammatory factors. This deserves to be followed up with further research.

Intrapancreatic fat deposition (IPFD) has received increasing attention in recent years. Excessive fat deposition in the pancreas raises the incidence of diabetic morbidity, pancreatitis and pancreatic cancer. IPFD can involve intralobular fat or interlobular fat, which affects the number of beta cells and promotes the development of diabetes or pancreatic exocrine disease, but the exact mechanism is not clear.^56^ Therefore, we will next conduct relevant studies to investigate whether FAK deficiency in pancreatic adipocytes has resulted in T1D.

**FIGURE 9:**
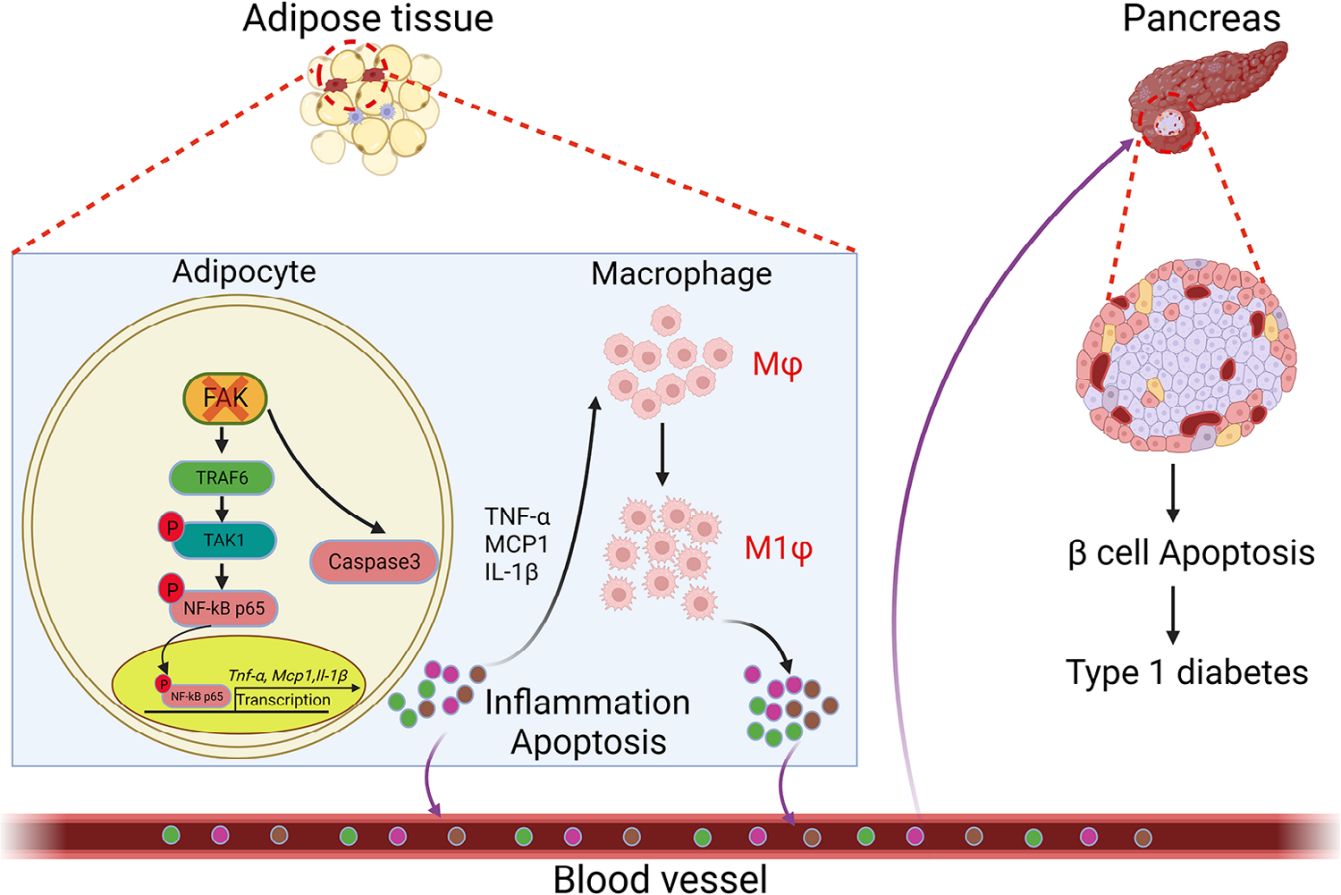
Schematic summary of the FAK‐mediated adipose tissue inflammation and pancreatic β cell apoptosis in diabetes. FAK deficiency in adipocytes activates the TRAF6/TAK1/NF‐κB signalling pathway as well as the activation of caspase3, which leads to inflammatory response as well as apoptosis in adipocytes, producing TNF‐α, IL‐1β and MCP1 inflammatory factors. These inflammatory factors further stimulate macrophages to polarise to M1 type, producing large amounts of inflammatory factors that circulate through the body and stimulate pancreatic β cell apoptosis, exacerbating the pathogenesis of diabetes.

In conclusion, Our findings demonstrated that FAK of AT plays a key role in the regulation of diabetes mellitus. Adipocyte FAK knockdown was able to activate TRAF6/TAK1/NF‐κB signalling pathway, producing inflammatory factors (TNF‐α, IL‐1β, MCP1). This opens a new avenue for activating inflammatory response pathways. These inflammatory factors further activate macrophages in adipose tissue to polarise to M1 type, and the inflammatory factors produced by M1‐type macrophages and adipocytes reach the pancreas through blood circulation, stimulate pancreatic β‐cell apoptosis and exacerbate the pathogenesis of STZ‐induced diabetes (Figure 9). Our data demonstrated relationship between FAK of AT and diabetes mellitus. Our research elaborate a new molecular mechanism for the involvement of AT in the regulation of diabetes, and may help develop new therapies for diabetes.

## AUTHOR CONTRIBUTIONS

F.D., H.J.C. and Y.E.Y. design and draft manuscripts. F.D., P.Z., H.T.F., Y.Y.L., Y.X.Y. and H.J.C. complete experiments. F.D. and Y.E.Y. analyse data and interpret. Y.E.Y. Write — Review & Edite, Fund acquisition. All authors revised and edited the paper.

## CONFLICT OF INTEREST STATEMENT

The authors declare no competing interests.

## FUNDING INFORMATION

This work was supported by grants to You‐e Yan (corresponding author) from the National Natural Science Foundation of China (No. 81970755 and 81570792), and the Large‐Scale Instrument and Equipment Sharing Foundation of Wuhan University.

## ETHICS STATEMENT

All animal studies were conducted following the Declaration of Helsinki and approved by the Animal Experiment Center of Wuhan University, which has been accredited by the International Association for the Evaluation and Accreditation of Laboratory Animal Care (AUP No. 20210529).

## Supporting information

Supporting Information

## Data Availability

The data that support the findings of this study are available from the corresponding author upon reasonable request.
