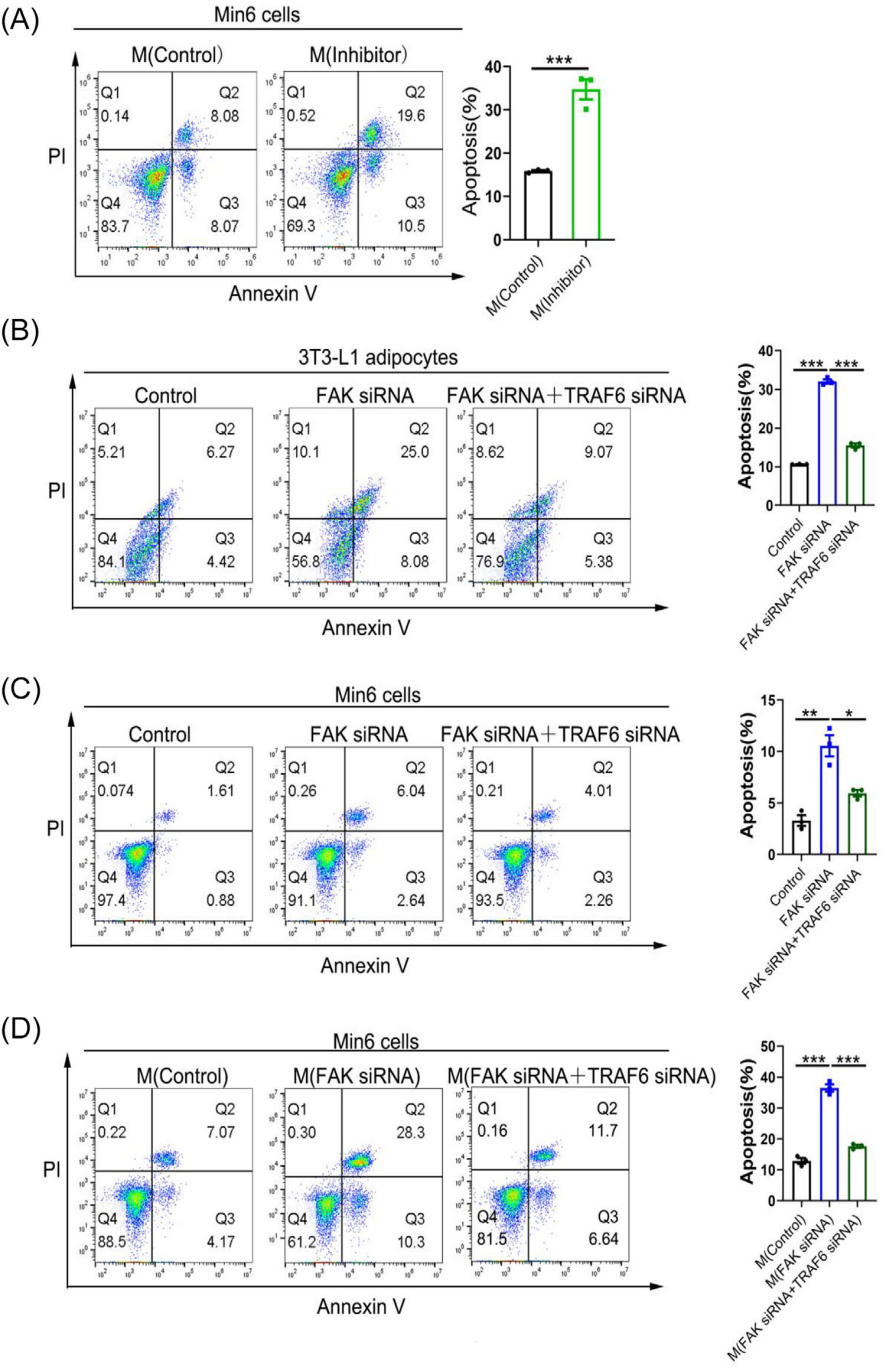# Corrigendum to ‘Adipocyte‐specific FAK deletion promotes pancreatic β‐cell apoptosis via adipose inflammatory response to exacerbate diabetes mellitus’

**DOI:** 10.1002/ctm2.70033

**Published:** 2024-09-24

In this article, we have just realized the wrong usage of flow cytometry chart in the FAK siRNA group in Figure S7B of Supplementary Material.

The wrong Figure S7 is as follows.


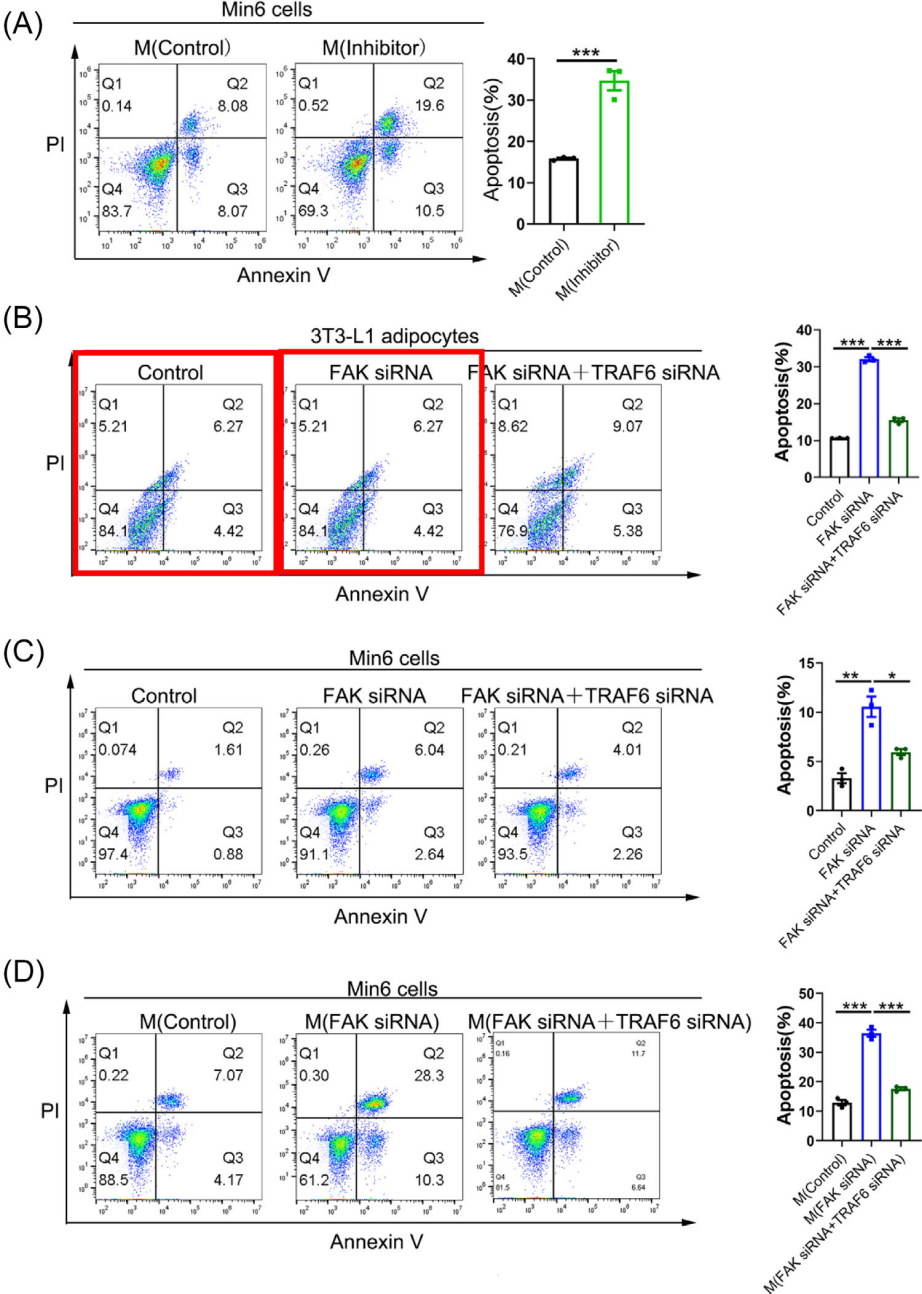


The corrected Figure S7 is as follows.