# Supplementary material for: Adipocyte‐specific FAK deletion promotes pancreatic β‐cell apoptosis via adipose inflammatory response to exacerbate diabetes mellitus
Source: Clin Transl Med. 2024 Jun 26;14(7):e1742. doi: 10.1002/ctm2.1742 (PMC11208094; doi:10.1002/ctm2.1742)
Supplement: Supplementary file 1 — Supporting Information [file CTM2-14-e1742-s001.docx]

**Supplementary Material**

Fig S1


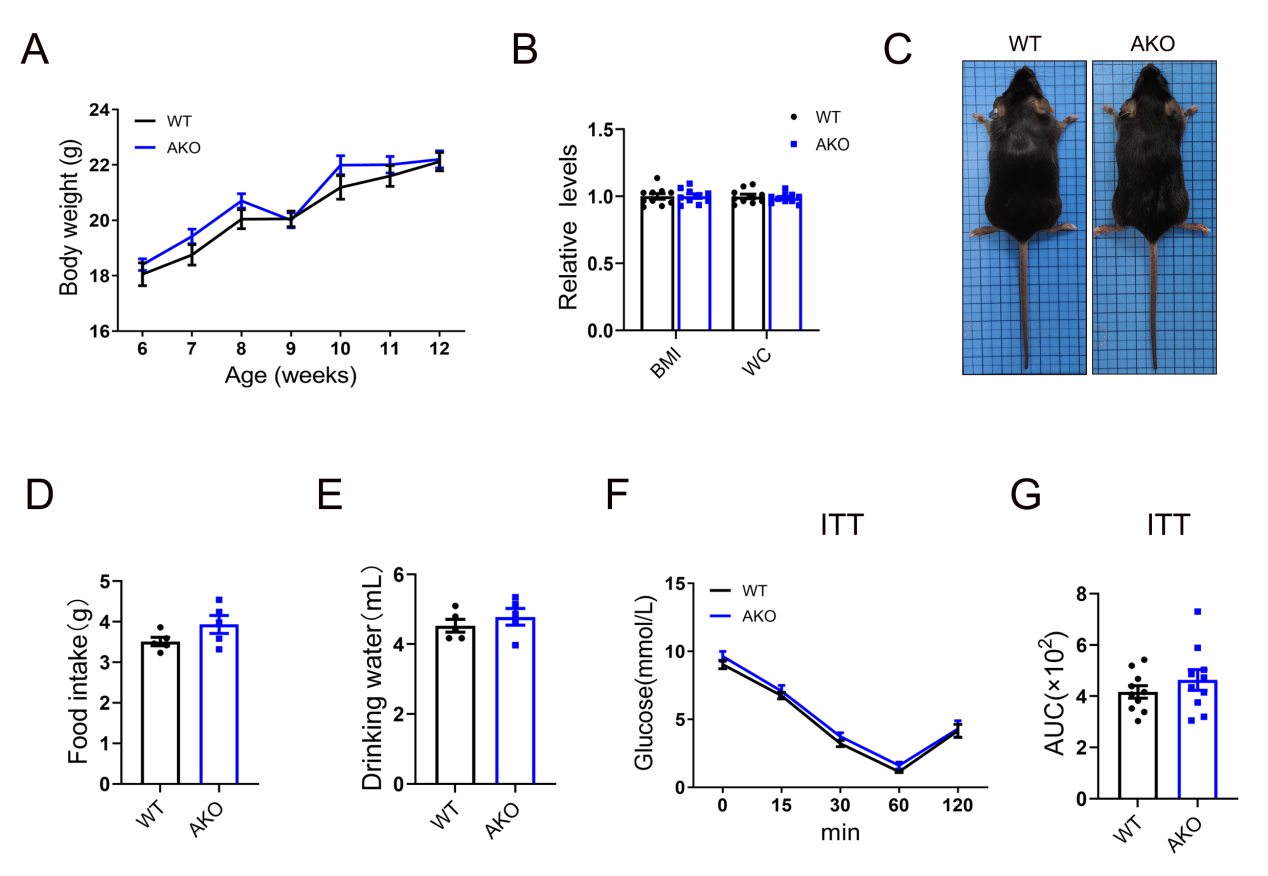


**Fig.S1** **Body weight and Insulin Tolerance Test(ITT) are not different between FAK AKO and WT mice.**

(A) Body weight of mice for 6 weeks(n = 10 per group). (B) BMI and WC in mice at 12 weeks of age (n = 10 per group). (C) macroscopic views of body size. (D-E) The average daily food intake and water intake per mice was monitored for five consecutive days at 10 weeks of age (n = 10 per group). (F-G) ITT and the AUC of ITT in mice at 11 weeks of age (n = 10 per group). BMI, Body Mass Index; WC, Waist Circumference; ITT, Insulin Tolerance Test. All values are expressed as MEAN ± SEM.

Fig S2


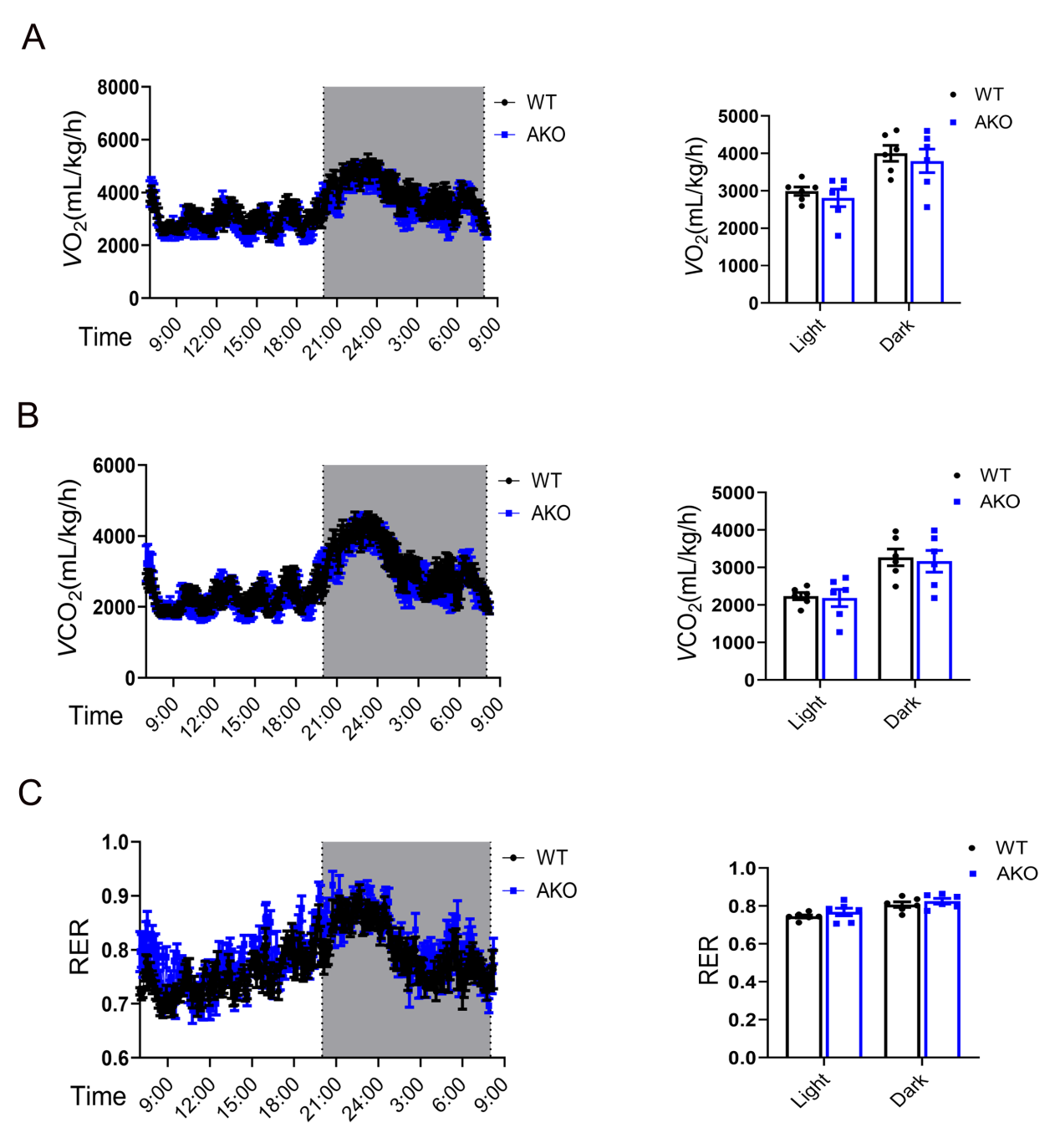


**Fig.S2 The body's energy metabolism is not different between FAK AKO and WT mice.**

C) CLAMS analysis *V*O_2_, *V*CO_2_ and RER in light and dark(n = 6 per group). CLAMS, Comprehensive Animal Metabolic Monitoring System; *V*O_2_, volumes of O2 consumption; *V*CO_2_, volumes of CO2 production; RER, respiratory exchange ratio. All values are expressed as MEAN ± SEM.

Fig S3


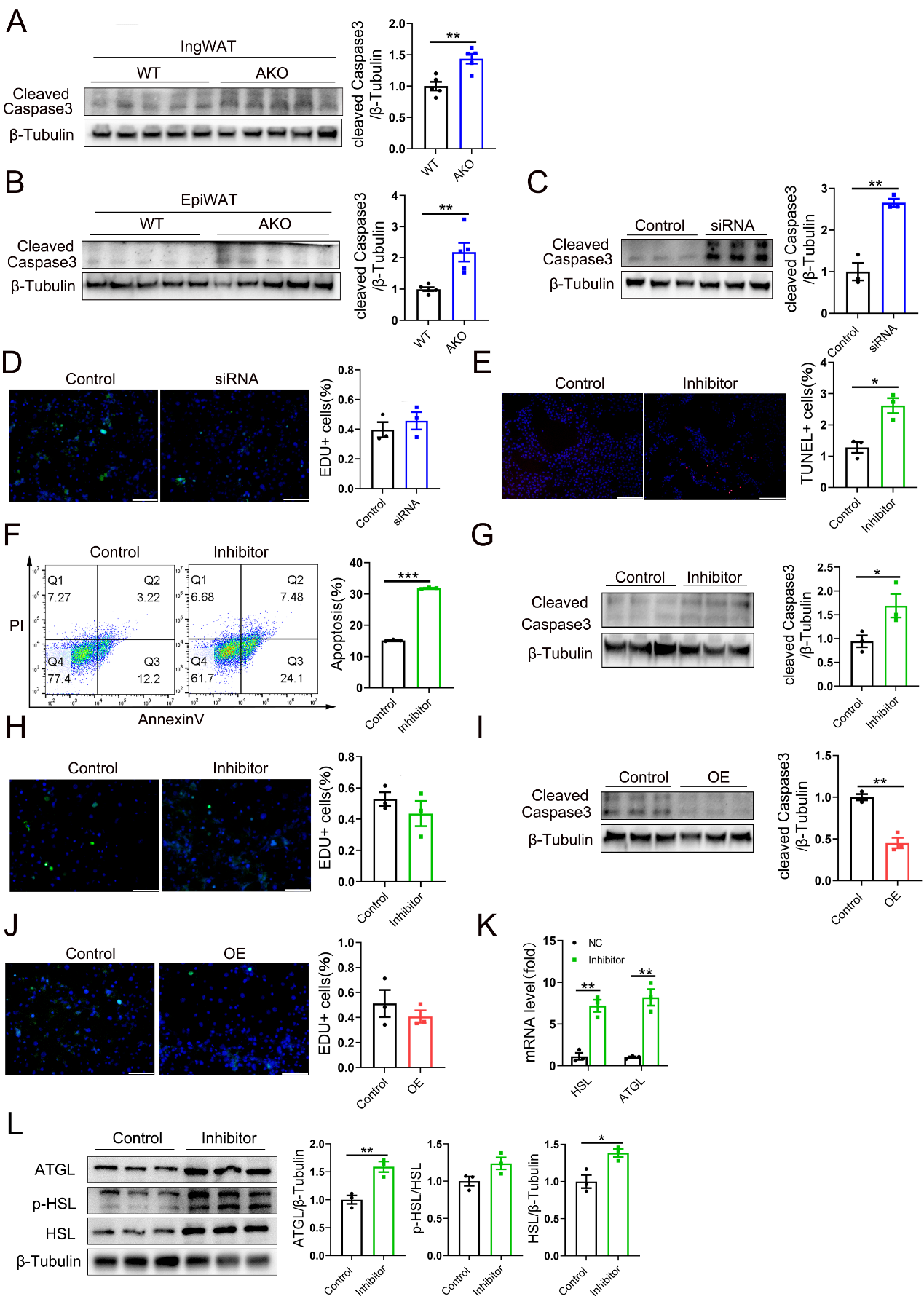


**Fig.S3 Adipocyte-specific FAK deletion does not affect adipocyte proliferation.**

(A-B) Western blot analysis protein expression of cleaved caspase3 in IngWAT and EpiWAT(n = 5 per group). (C, G, I) Western blot analysis protein expression of cleaved caspase3 in FAK siRNA group, FAK inhibitor group, FAK OE group(n = 3 per group). (D, H, J) Fluorescence microscope images analysis the EDU+ cells in FAK siRNA group, FAK inhibitor group, FAK OE group(n = 3 per group). Scale bar, 100 μm. (E) Fluorescence microscope images analysis the TUNEL+ cells in FAK inhibitor group(n = 3 per group). Scale bar, 100 μm. (F) Flow cytometry analysis the number of apoptosis cells in FAK inhibitor group(n = 3 per group). (K) qPCR analysis mRNA expression of HSL and ATGL in FAK inhibitor group(n = 3 per group). (L) Western blot analysis protein expression of HSL, p-HSL and ATGL in FAK inhibitor group (n = 3 per group). IngWAT*,* inguinal white adipose tissue; EpiWAT, epididymal white adipose tissue; HSL, hormone-sensitive lipase; ATGL, adipose triglyceride lipase. All values are expressed as MEAN ± SEM**,** **P* < 0.05**,** ***P* < 0.01**,** ****P*< 0.001.

Fig S4


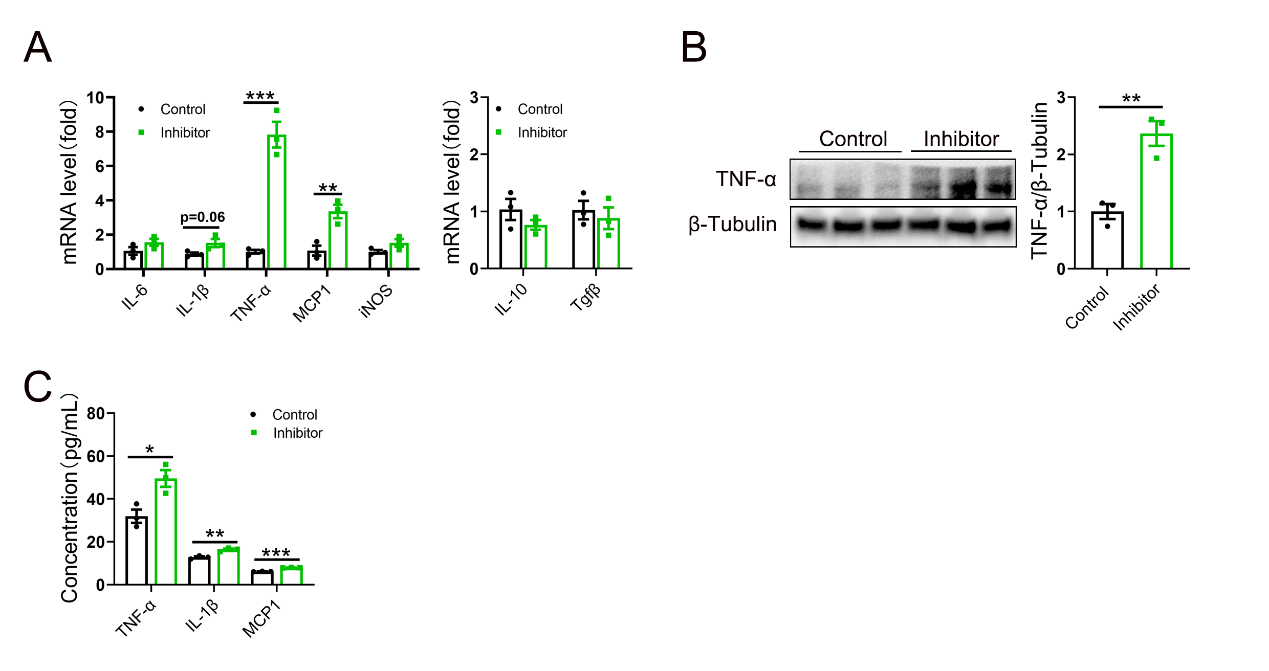


**Fig.S4 Inhibition of FAK activity promotes adipocyte inflammatory response.**

1. qPCR analysis mRNA expression of IL-6, IL-1β, TNF-α, MCP1, iNOS, IL-10, Tgfβ in FAK inhibitor group(n =3 per group). (B) Western blot analysis protein expression of TNF-α in FAK inhibitor group(n =3 per group). (C) ELISA analysis TNF-α, IL-1β, MCP1 concentration in the supernatant of FAK inhibitor group(n =3 per group). All values are expressed as MEAN ± SEM**,** **P* < 0.05**,** ***P* < 0.01**,** ****P*< 0.001.

Fig S5


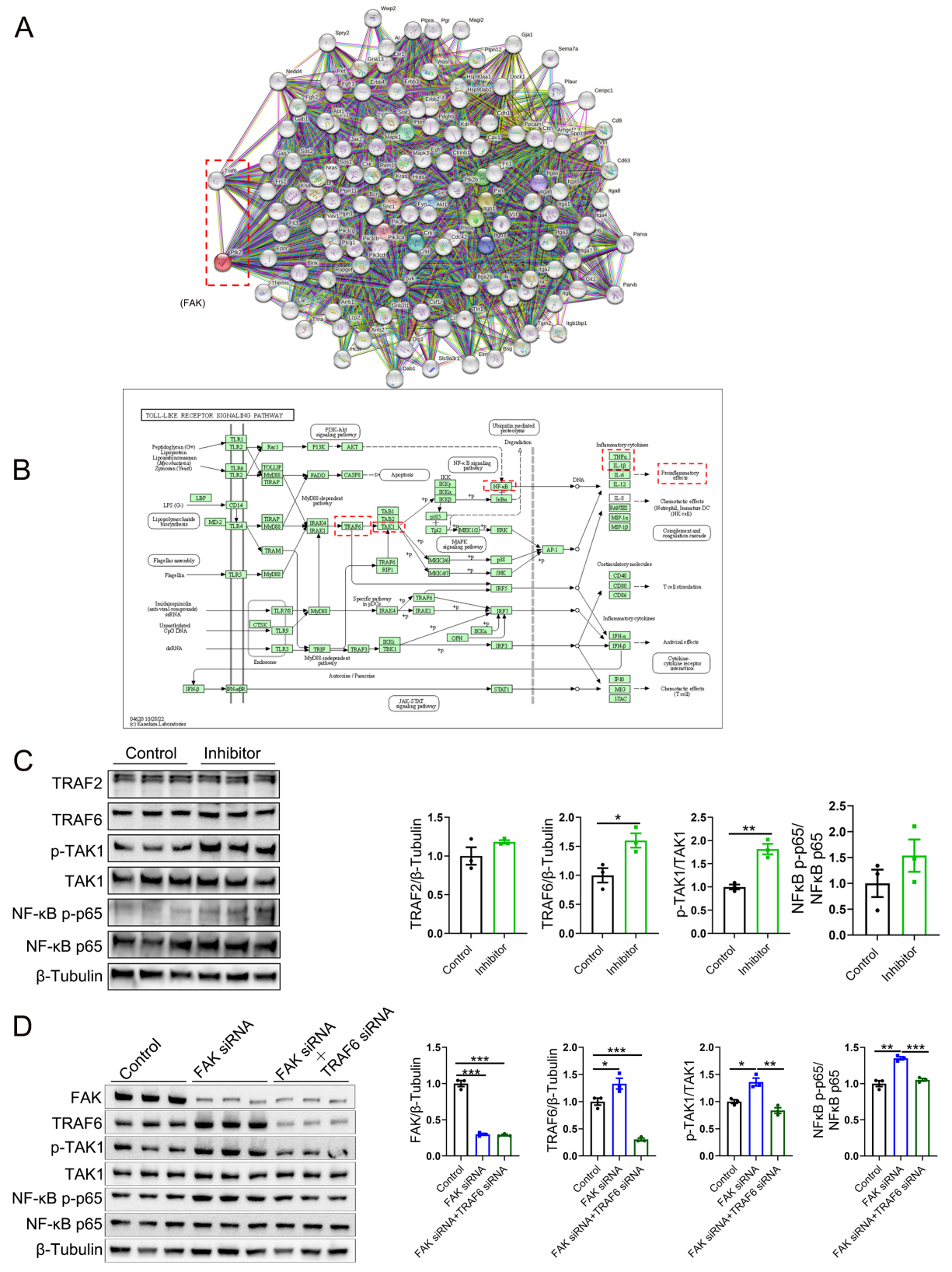


**Fig.S5 Inhibition of FAK activity exacerbates adipocyte inflammatory response through activation of TRAF6/TAK1/NF-κB signaling pathway.**

1. Using the STRING Website, predicting proteins that interact with FAK and constructing PPI

network. (B) The signaling pathways enriched with FAK-interacting proteins were analyzed and the most relevant signaling pathways to inflammation were selected for validation. (C) Western blot analysis protein expression of TRAF2, TRAF6, TAK1, p-TAK1, NF-κB p65, NF-κB p-p65 in FAK inhibitor group(n =3 per group). (D) Western blot analysis protein expression of FAK, TRAF6, TAK1, p-TAK1, NF-κB p65, NF-κB p-p65 in FAK siRNA, combining FAK siRNA with TRAF6 siRNA group(n =3 per group).PPI, Protein–protein interaction; All values are expressed as MEAN ± SEM**,** **P* < 0.05**,** ***P* < 0.01**,** ****P*< 0.001.

Fig S6


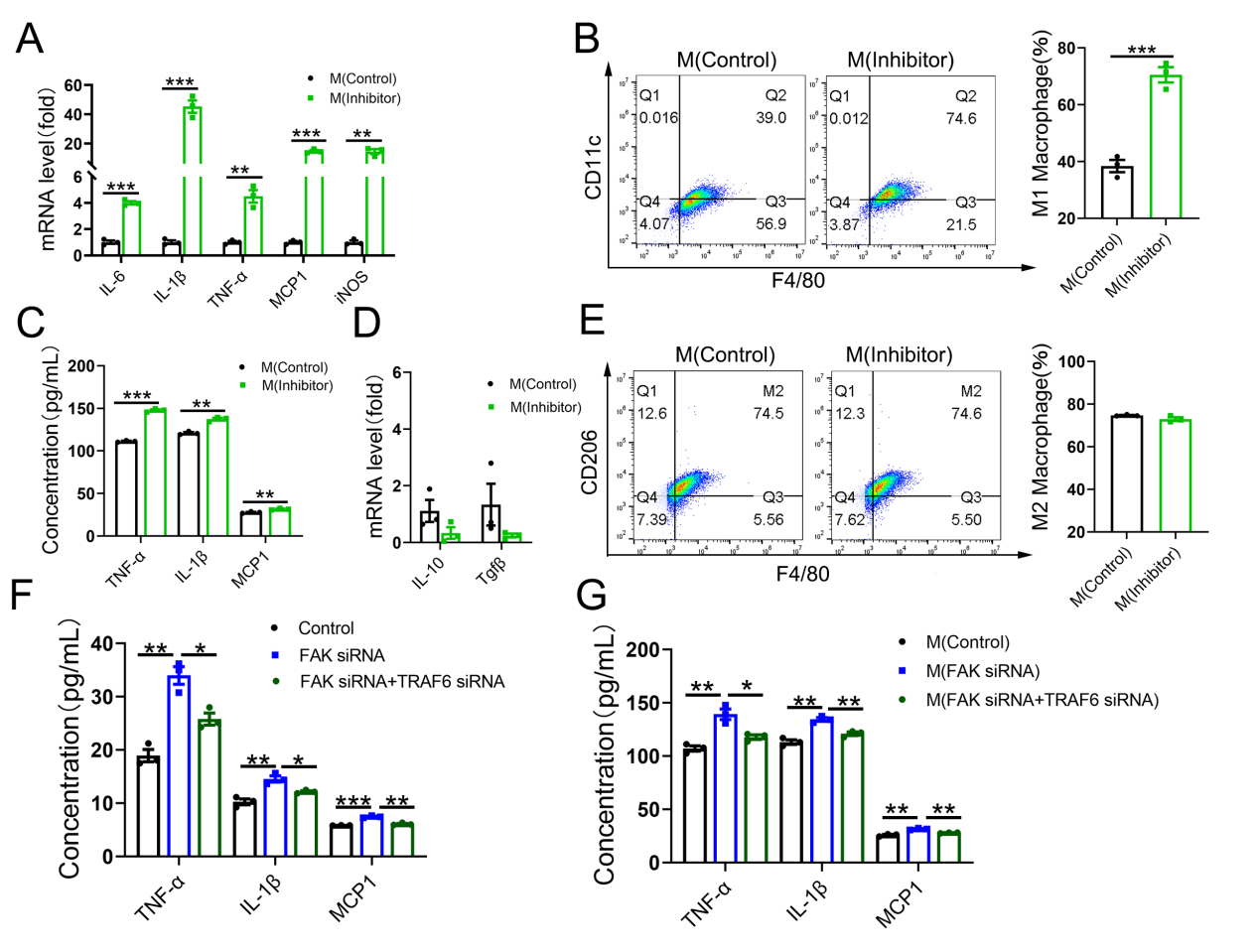


**Fig.S6 FAK regulates cytokine secretion primarily by acting on the TRAF6 pathway.**

Macrophages were co-cultured with adipocytes（FAK inhibitor treatment）and the addition of LPS stimulation for 48h(A-C). (A) qPCR analysis mRNA expression of IL-6, IL-1β, TNF-α, MCP1, iNOS in RAW264.7 cells(n =3 per group). (B) Flow cytometry analysis the number of M1 macrophages in RAW264.7 cells(n = 3 per group). (C) ELISA analysis TNF-α, IL-1β, MCP1 concentration in the supernatant of RAW264.7 and 3T3-L1 adipocyte cells(n =3 per group). Macrophages were co-cultured with adipocytes（FAK inhibitor treatment）and the addition of IL-4 stimulation for 48h(D-E). (D) qPCR analysis mRNA expression of IL-10, Tgfβ in RAW264.7 cells(n =3 per group). (E) Flow cytometry analysis the number of M2 macrophages in RAW264.7 cells(n = 3 per group). (F) ELISA analysis TNF-α, IL-1β, MCP1 concentration in the supernatant of 3T3-L1 adipocyte cells(n =3 per group). (G) ELISA analysis TNF-α, IL-1β, MCP1 concentration in the supernatant of RAW264.7 and 3T3-L1 adipocyte cells (n =3 per group). All values are expressed as MEAN ± SEM**,** **P* < 0.05**,** ***P* < 0.01**,** ****P*< 0.001.

Fig S7


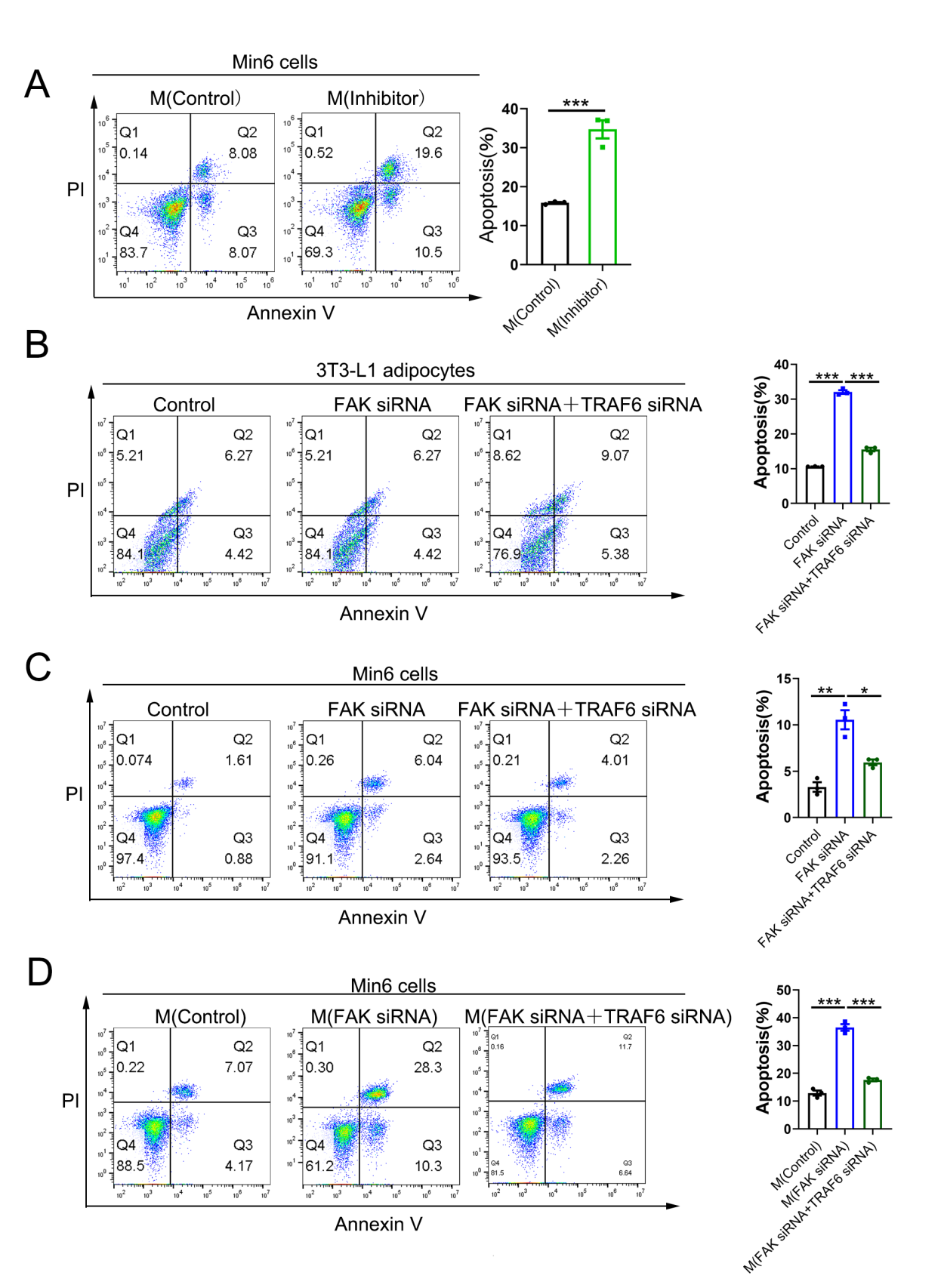


**Fig.S7 FAK regulates pancreatic β-cell apoptosis primarily by acting on the TRAF6 pathway.**

(A,D) Supernatant collected from co-culture of adipocytes(FAK inhibitor, FAK siRNA, FAK siRNA with TRAF6 siRNA) and macrophages(LPS stimulation) was treated with min6 cells for 48h, Flow cytometry analysis the number of apoptosis cells(n = 3 per group). (B) Flow cytometry analysis the number of apoptosis adipocytes in FAK siRNA, FAK siRNA with TRAF6 siRNA group(n = 3 per group). (C) Supernatant collected from adipocytes(FAK siRNA, FAK siRNA with TRAF6 siRNA) was treated with min6 cells for 48h, Flow cytometry analysis the number of apoptosis cells(n = 3 per group). All values are expressed as MEAN ± SEM**,** ****P*< 0.001.

Fig S8


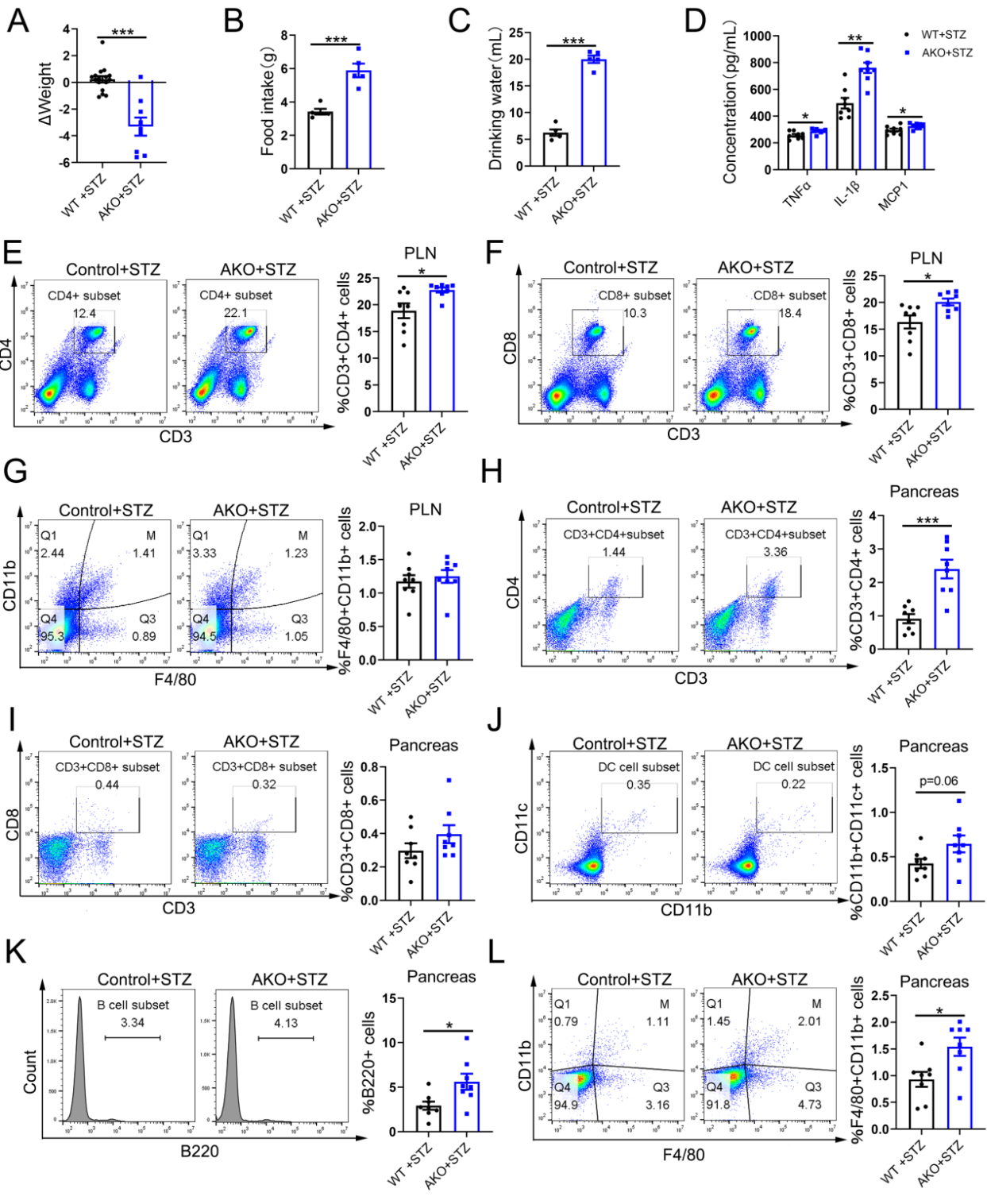


**Fig.S8 Adipocyte-specific FAK deletion exacerbates the inflammatory response in PLN and pancreas of diabets mice.**

Mice were injected with 40mg/kg/day of STZ for five consecutive days. (A) Body weight changes of mice 28 days after STZ injection(nWT=16, nAKO=9). (B-C) The average daily food intake and water intake per mice was monitored for five consecutive days at 15 days after STZ injection(nWT=16, nAKO=9). (D) ELISA analysis serum TNF-α, IL-1β, MCP1 concentration (n = 8 per group). (E-G) Flow cytometry analysis the number of CD4+T, CD8+T, Macrophage cells in PLN(n = 8 per group). (H-L) Flow cytometry analysis the number of CD4+T, CD8+T, Macrophage, DC, B cells in pancreas(n = 8 per group). ΔWeight, difference in weight at the end of modeling and before modeling. PLN, Pancreatic lymph nodes. All values are expressed as MEAN ± SEM**,** **P* < 0.05**,** ***P* < 0.01**,** ****P*< 0.001.

**Table S1. Primers sequences for real-time quantitative PCR**

| Genes | Forward primer | Reverse primer | Spe. | Pro. |
| --- | --- | --- | --- | --- |
| FAK(ptk2） | GAGTACGTCCCTATGGTGAAGG | CTCGATCTCTCGATGAGTGCT | Mus | 108 |
| HSL | GGCTCACAGTTACCATCTCACC | GAGTACCTTGCTGTCCTGTCC | Mus | 107 |
| ATGL | GAGACCAAGTGGAACATC | GTAGATGTGAGTGGCGTT | Mus | 120 |
| IL-6 | TCCTACCCCAATTTCCAATGCT | TAACGCACTAGGTTTGCCGA | Mus | 149 |
| IL-1β | TGCCACCTTTTGACAGTGATG | ATGTGCTGCTGCGAGATTTG | Mus | 136 |
| TNF-α | ATGGCCTCCCTCTCATCAGT | TTTGCTACGACGTGGGCTAC | Mus | 97 |
| MCP1 | TGCCCTAAGGTCTTCAGCAC | AAGGCATCACAGTCCGAGTC | Mus | 150 |
| iNOS | ATTAGCCAGAAGGGTCCCTGA | GCGCTCCCAGGAAATGTTTT | Mus | 81 |
| IL-10 | GCTCTTACTGACTGGCATGAG | CGCAGCTCTAGGAGCATGTG | Mus | 105 |
| Tgfβ | AATGGTGGACCGCAACAAC | GCACTGCTTCCCGAATGTC | Mus | 98 |
| Rplp0 | TGGCTGATCCATCTGCATTT | GATTCCTCCGACTCTTCCTTTG | Mus | 115 |

Spe. - Species; Pro. - Product (bp).
